# RPA hyperphosphorylation hinders the resolution of R-loops and G-quadruplex-associated R-loops during RAS-driven senescence

**DOI:** 10.1093/nar/gkag331

**Published:** 2026-04-13

**Authors:** Ylenia Cortolezzis, Vanessa Tolotto, Luca Triboli, Raffaella Picco, Miguel A Soler, Sara Fortuna, Giacomo Bettin, Francesca D’Este, Enrico Carlassara, Gabriele Magris, Kyle M Miller, Alessandro Angelini, Luigi E Xodo, Eros Di Giorgio

**Affiliations:** Laboratory of Biochemistry, Department of Medicine, University of Udine, Piazzale M. Kolbe 4, 33100 Udine, Italy; Laboratory of Biochemistry, Department of Medicine, University of Udine, Piazzale M. Kolbe 4, 33100 Udine, Italy; Laboratory of Biochemistry, Department of Medicine, University of Udine, Piazzale M. Kolbe 4, 33100 Udine, Italy; Laboratory of Biochemistry, Department of Medicine, University of Udine, Piazzale M. Kolbe 4, 33100 Udine, Italy; Dipartimento di Scienze Matematiche, Informatiche e Fisiche, University of Udine, Via delle Scienze 206, 33100 Udine, Italy; Department of Molecular Sciences and Nanosystems, Ca' Foscari University of Venice, Via Torino 155, 30172 Mestre, Italy; Dipartimento di Scienze Matematiche, Informatiche e Fisiche, University of Udine, Via delle Scienze 206, 33100 Udine, Italy; Laboratory of Biochemistry, Department of Medicine, University of Udine, Piazzale M. Kolbe 4, 33100 Udine, Italy; Department of Molecular Sciences and Nanosystems, Ca' Foscari University of Venice, Via Torino 155, 30172 Mestre, Italy; Laboratory of Biochemistry, Department of Medicine, University of Udine, Piazzale M. Kolbe 4, 33100 Udine, Italy; Laboratory of Biochemistry, Department of Medicine, University of Udine, Piazzale M. Kolbe 4, 33100 Udine, Italy; Department of Agricultural, Food, Environmental and Animal Sciences, University of Udine, 33100 Udine, Italy; Department of Radiation Oncology, Emory University School of Medicine, Winship Cancer Institute, GA 30307 Atlanta, United States; Department of Molecular Sciences and Nanosystems, Ca' Foscari University of Venice, Via Torino 155, 30172 Mestre, Italy; Laboratory of Biochemistry, Department of Medicine, University of Udine, Piazzale M. Kolbe 4, 33100 Udine, Italy; Laboratory of Biochemistry, Department of Medicine, University of Udine, Piazzale M. Kolbe 4, 33100 Udine, Italy

## Abstract

Activation of RAS oncogenes in normal cells triggers a stable cell cycle arrest known as RAS-induced senescence (RIS), marked by persistent DNA damage and extensive epigenetic remodeling. Although bypassing RIS promotes tumorigenesis, the molecular mechanisms underlying this transition remain poorly defined. Here, we demonstrate that RIS cells accumulate high levels of R-loops—three-stranded DNA–RNA hybrids—that frequently co-localize with DNA G-quadruplexes formed on the non-template DNA strand, generating G-loop-like structures. RIS bypass is characterized by the resolution of these structures through the heterotrimeric RPA complex, which facilitates RNase H1-mediated R-loop processing. In pre-RIS and RIS cells, hyperphosphorylation of RPA32 disrupts the ability of RPA to enhance RNase H1 activity, thereby impairing its enzymatic processivity. Consequently, R-loops and G-loops remain unresolved, contributing to the accumulation of γH2AX. Remarkably, forced restoration of RPA-regulated RNase H1 activity in RAS-expressing cells reduces DNA damage and enables cell cycle re-entry, effectively bypassing senescence. These findings identify a regulatory axis involving RPA phosphorylation and RNase H1 activity that governs R-loop and G-loop resolution, acting as a critical genome maintenance mechanism during oncogene-induced stress.

## Graphical Abstract

**Graphical Abstract:**
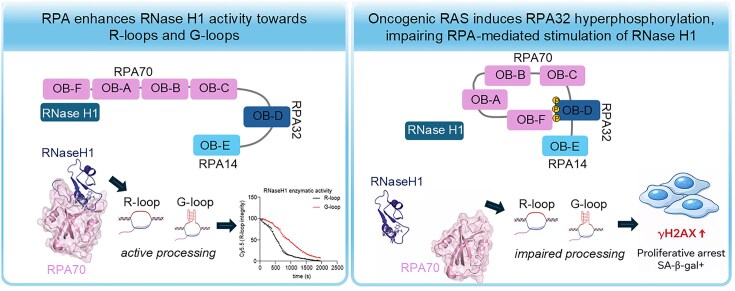

## Introduction

Aberrant oncogene activation in primary healthy cells leads to oncogene-induced senescence (OIS) [1]. OIS cells accumulate in various pre-malignant lesions to halt malignant transformation [2–5] and in chemotherapy-treated tumors [6]. However, OIS cells remain metabolically active and acquire a highly secretory phenotype (SASP, senescence-associated secretory phenotype) by releasing soluble and insoluble pro-inflammatory factors into the extracellular environment, exerting harmful paracrine pro-tumoral effects on nearby cells [7]. Among the various forms of OIS, RAS-induced senescence (RIS) is the most extensively studied [1, 2]. In RIS cells, cell cycle arrest is maintained by the p53 and RB tumor suppressor pathways, which are activated in response to DNA damage triggered by replication stress following oncogenic RAS activation [8, 9]. However, it is reported that blockade of DNA-damage response (DDR) or tumor suppressors like p53 and RB, once OIS is well established, is not sufficient to escape RIS [8, 10–12]. Conversely, a change in epigenetic balance in cells subjected to replication stress following oncogenic activation can lead to RIS bypass [13–15]. This has led to the search for epigenetic markers that can determine the initial stages of tumorigenesis, although the impact of RIS evasion on neoplastic transformation has yet to be uncovered [16, 17]. HDAC4 is a class IIa histone deacetylase that is catalytically inactive but able to interact with class I HDACs—most prominently HDAC3—thereby modulating chromatin acetylation at specific genomic loci [18]. During OIS, HDAC4 is selectively degraded by the ubiquitin–proteasome system [13]. Conversely, its overexpression allows bypass of senescence [19] by coordinating two epigenetic processes essential for the senescence program [13, 20]. First, HDAC4 restrains H3K27ac levels at a subset of super–enhancers that drive the transcriptional activation of SASP genes, thereby preventing the enhancer hyperacetylation that normally accompanies OIS [13]. Second, HDAC4 contributes to the regulation of the chromatin response to DNA damage by controlling the switch from H2BK120 acetylation to H2BK120 ubiquitination at DNA damaged sites [20].

Large-scale epigenetic modifications have been described during OIS [21–23], including the formation of non-B DNA secondary structures that have been reported to promote replication fork stalling and DDR activation [24, 25]. Among non-B DNA structures, R-loops, consisting of a chimeric DNA:RNA double helix and a displaced complementary single-stranded DNA, have been found to increase in cellular senescence [26–28]. Aberrant accumulation of R-loops was reported to trigger DNA replication stress [29], sustain cellular senescence [30] and contribute to the release of immunogenic single-stranded DNAs in the cytoplasm [31, 32]. At the level of individual loci, the processing of R-loops occurs by specific helicases or ribonucleases (reviewed in ref. [33]). If not recognized and resolved, aberrant accumulation of R-loops increases genomic instability in three ways: (i) in S-phase, by exacerbating transcription-replication conflicts leading to fork stalling and DSBs generation [34]; (ii) outside S-phase, two consecutive R-loops can be processed by nucleotide excision repair (NER) nucleases XPG and XPF leading to the formation of DSBs [35], while the displaced ssDNA of R-loops is prone to accumulate single-strand breaks resulting from the exposure to reactive oxygen species or by the action of BER glycosylase after cytidine deamination (reviewed in [36]); (iii) during DSB repair, by interfering with end resection and homologous recombination [37–39]. In contrast, R-loop formation near DSBs in actively transcribed loci is reported to directly support the RAD52-mediated transcription-associated homologous recombination directed repair (HDR) [40] or the RAD51AP1/UAF1 dependent DR-loop formation and homologous recombination repair [41]. To allow the maintenance of cellular fitness, R-loops processing is tightly regulated through the control of RNA modification and RNA editing [42] or the locus-specific recruitment of R-loop resolvases [33].

The frequent co-occurrence of R-loops and DNA G-quadruplexes (G4s) has led to the hypothesis that hybrid structures may form, integrating an R-loop with a G4 structure on the displaced DNA strand [36, 43]. These structures, termed G-loops, have recently been identified and appear to arise not from *de novo* transcription [44], but rather through homology-directed RNA invasion of the DNA strand complementary to the G4 [45]. G-loops may therefore represent a transient intermediate during the molecular resolution of G-quadruplexes [45]. However, the molecular events that follow G4 resolution, as well as the precise mechanisms underlying R-loop processing, remain poorly understood. Equally unclear are the consequences of defective resolution, which may lead to persistent genome instability and replication stress.

It is known that the integration of multiple regulatory mechanisms ensures the correct metabolism of R-loops [42, 45]. Failure of one or more of these processing mechanisms has been observed in precancerous lesions, leading to the accumulation of R-loops and genome instability [46, 47]. Elevated levels of R-loops have been observed in cells undergoing OIS; in fact, despite the high expression of RNase H1, the enzyme appears unable to efficiently resolve these structures in RAS-expressing cells [28]. However, overexpression of RNase H1 or RNase H2 was effective in partially reducing R-loop accumulation and alleviate replication stress, suggesting that increased enzymatic activity can restore genome stability under conditions of excessive R-loop formation [28, 47].

In this study, we demonstrate that RIS is characterized by elevated levels of R-loops, which frequently co-occur with G4s on the displaced DNA strand, resulting in the formation of G-loop-like structures. We show that the replication protein A (RPA) complex [48, 49] plays a key role in preserving genome integrity by promoting RNase H1-mediated processing of R-loops and G-loops. In RIS cells, however, RPA32 undergoes hyperphosphorylation—a hallmark of oncogenic stress—which alters the conformation of the RPA complex and impairs its interaction with RNase H1. This disruption compromises RNase H1 processivity, leading to inefficient resolution of DNA:RNA hybrids and G4-associated structures. The resulting accumulation of unresolved R-loops and G-loops is associated with sustained γH2AX signal, reinforcing the senescent phenotype.

## Materials and methods

### Cell lines and reagents

BJ-TERT cells (RRID: CVCL_C8QN) were obtained from ATCC and grown in Earle’s salts Minimal Essential medium (MEM) containing 1.5 g/l glucose (Euroclone, Italy) supplemented with 10% fetal bovine serum (Capricorn Scientific, South American), 2 mM l-glutamine, 1 mM pyruvate, 1x MEM nonessential amino acids, 10 units/ml penicillin and 10 μg/ml streptomycin (all from Euroclone, Italy) at 37°C and 5% CO_2_. HEK-293 (RRID: CVCL_9867), IMR90 (RRID: CVCL_0347), Phoenix-Ampho (RRID: CVCL_H716), U2OS (RRID: CVCL_0042) and PANC-1 cells (RRID:CVCL_0480) cells were obtained from American Type Culture Collection (ATCC, USA). U2OS-LacO-I-SceI-TetO were obtained from Kerafast (US). These cell lines were maintained in DMEM culture medium (Euroclone, Italy) containing 4.5 g/l glucose (Euroclone, Italy) supplemented with 10% fetal bovine serum (Capricorn Scientific, South American), 2 mM l-glutamine, 10 units/ml penicillin and 10 μg/ml streptomycin (all from Euroclone, Italy) at 37°C and 5% CO_2_. BJ/hTERT, BJ/hTERT/HRAS^G12V^-ER, and BJ-hTERT/HRAS^G12V^-ER/HDAC4-FLAG cells were previously characterized [13, 20]. Regular testing for Mycoplasma contamination was performed on all cell lines.

The following chemical reagents were used: 1 µM 4-hydroxytamoxifen (4-OHT), Trypan Blue, 12 µM Camptothecin (CPT) (Enzo Life Sciences Farmingdale, NY, USA), 1 µM Doxycycline, 10 µM NSC15520, Puromycin (0.5 µg/ml), and 50 µM Bromodeoxyuridine (BrdU) (all from Sigma-Aldrich, Germany).

### Plasmid construction

Plasmids including RED (#139835), dRED (#139836), pLenti-CMV/TO-GFP-MDC1 (#26285), pQCXIP-GFP-RPA70 (#164231), p11d-tRPA(123) (#102613), pcDNA3-Flag-RPA32 (#22893), p11d-tRPA-32Asp8 (#102617), p11d-tRPA-32Ala9 (#102616) and pTK168_mCherry-H2B (#46363) were obtained from Addgene (USA). pEGFP-N1, pCDNA3, pBABE-H2B-GFP, pFLAG-CMV5, pGEX-4T1 and pET32a + were obtained by collaboration with prof. Claudio Brancolini (University of Udine, Italy).

pEGFP-N1-RNase H1, pEGFP-N1-dRNase H1, pFLAG-CMV5a-RNase H1, pFLAG-CMV5a-dRNase H1, pCDNA3-RNase H1-EGFP-dCas9, pCDNA3-dRNase H1-EGFP-dCas9, pDEST-mCherry-LacR-mCherry, pDEST-mCherry-LacR-RNase H1, pDEST-mCherry-LacR-dRNase H1, pTK168_mCherry- RNase H1, pEGFP-C1/pEBFP-C1-RPA32 WT, pEGFP-C1/pEBFP-C1-RPA32 Ala9, pEGFP-C1/pEBFP-C1-RPA32 Asp8, pEGFP-N1-RPA70 1-101, pEGFP-N1-RPA70 and deletion mutants, pGEX-4T1-FLAG-RPA70 deletion mutants, pGEX-4T1-RPA70, pET32a+-RNase H1 were generated with a PCR-restriction based methods and controlled by sequencing. Mutants of RPA32 (RPA32 S4/8A, RPA32 S33A, RPA32 S4/8A + S33A) were cloned into pEGFP-C1 and pEBFP-C1 starting from the p11d-tRPA-32Ala9 (#102616) plasmid. Destination vectors were digested with BglII/SalI, and insert products were digested with BamHI/SalI restriction enzymes.

pCDNA3-FLAG-RPA70 1-27, pCDNA3-FLAG-RPA70 27-47, pCDNA3-FLAG-RPA70 56-70 and pCDNA3-FLAG-RPA70 71-96 were generated through oligo-cloning, involving digestion of the destination vector pcDNA3-Flag-RPA32 with BamHI/XbaI enzymes, oligo phosphorylation, annealing and ligation. pCW-Puro- GFP-(d)RNase H1 was generated by subcloning the CDS of RED (#139835) and dRED (#139836) in pCW-Puro backbone.

pCMV-3xNLS-I-SceI was kindly provided by Prof. Fabrizio D’Adda di Fagagna (IFOM Foundation, FIRC Institute of Molecular Oncology Foundation, Milan, Italy).

### Cell transfection and retroviral infection

293T and Ampho cells were transfected using polyethylenimine (PEI, 1 μg/ml) at a 2:1 ratio of PEI (μl) to DNA (μg). Transient expression in U2OS and BJ cells was achieved via Lipofectamine 2000 (Thermo Fisher, Waltman, USA), following manufacturer’s specifications. For retroviral infections, Ampho cells were seeded in 60 mm dishes and transfected with 10 µg retroviral plasmids. For lentiviral infections, 293T cells were seeded in 60 mm dishes and transfected with 1.8 µg VSV-G, 5 µg Δ8.9 and 12 µg of lentiviral plasmids. After incubation at 32°C (retroviral) or 37°C (lentiviral), virions were collected, filtered through 0.45 µm filter, diluted 1:1 in fresh medium and used to infect BJ or IMR90 cells. Uninfected cells were subjected to negative selection using Puromycin (0.5 µg/ml), Hygromycin B (150 µg/ml), Blasticidin (8 µg/ml) or G418 (300 µg/ml) for two days.

### Senescence-associated beta-galactosidase (SA-β-Gal) assay

Cells seeded on coverslips in 12-well plates were fixed for 5′ (PBS 2% formaldehyde/0.2% glutaraldehyde), washed twice with 0.9% NaCl, and stained for 16 h at 37 °C with staining solution: 40 mM citric acid/Na phosphate buffer, 5 mM K4[Fe (CN)6]3H2O, 5 mM K3[Fe (CN)6], 150 mM sodium chloride, 2 mM magnesium chloride, and 1 mg/mL X-gal (Panreact Applichem, Italy). Images were acquired with Leica LD bright field optical microscope.

### Co-immunoprecipitation

After culturing for the specified time under the described conditions, cells were washed twice with PBS and harvested with hypotonic Low Salt Buffer (200 mM Tris–HCl pH 7.5, 1% Triton X-100, 10 mM MgCl2, 10 mM KCl, 1% glycerol) freshly supplemented with protease inhibitor cocktail (PIC), 1 mM PMSF, 1 mM NaVO4 and 1 mM NaF. Cells were lysed on ice for 10 min and 150 mM NaCl was added to stop the lysis. The nuclei were lysed with an insulin syringe and the samples were centrifuged. The supernatant was pre-cleared for at least 1 h on a rotating mixer at 4°C. Dynabeads Protein A (Thermo Fisher Scientific, USA) was used for immunoprecipitation with anti-rabbit antibodies, while Dynabeads Protein G (Thermo Fisher Scientific) was used for anti-mouse antibodies. Inputs were collected and samples were incubated with 1 µg of the indicated primary antibody (FLAG M2, GFP, RPA70, RNase H1) overnight on a rotating mixer at 4°C. Immunoprecipitated complexes were collected with 8 µl of Dynabeads for 2 h. Samples were washed three times with High Salt Buffer (200 mM Tris–HCl pH 7.5, 150 mM NaCl, 1% Triton X-100, 10 mM MgCl2, 10 mM KCl, 2 mM EDTA, 1% glycerol) and 2X Laemmli buffer was added before boiling for 5 min and subjected to SDS-page and immunoblotting.

### Nucleic acids extraction, reverse transcription and qPCR

Genomic DNA (gDNA) was extracted using the Quick-DNA Miniprep Kit (Zymo Research), following the manufacturer’s instructions. Total RNA was extracted following acid guanidinium thiocyanate-phenol-chloroform extraction method (Tri Reagent, Molecular Research Center) 1.0 μg of total RNA was DNAseI treated (NEB #T2010) and reverse-transcribed by using 100 units of M-MLV Reverse transcriptase (Life Technologies, USA) in the presence of 1.6 μM oligo(dT) (Sigma-Aldrich) and 4 μM Random hexamers (Euroclone, Italy). qRT-PCRs were performed using SYBR green technology (KAPA Biosystems). qPCR of gDNA was performed by using 2.5 ng gDNA as template. Data were analyzed by comparative threshold cycle (delta delta Ct) using HPRT, GAPDH as normalizers for qRT-PCR, or ACTB and GAPDH as qPCR normalizers. All reactions were carried out in triplicate. The sequence of used primers is provided in Supplementary Table S1.

### Immunofluorescence and immunoblotting

Immunofluorescence was performed as previously described [13]. Briefly, cells were fixed with 3% paraformaldehyde and permeabilized with 0.3% Triton X-100. The secondary antibodies were Alexa Fluor 488-, 532- 546- or 633-conjugated anti-mouse and anti-rabbit secondary antibodies (Molecular Probes). For S phase analysis, cells were grown for 3 h with 50 μM Bromodeoxyuridine (BrdU, Sigma-Aldrich). After fixation, coverslips were treated with HCl (1% and 2%), quenched with borate buffer, and processed for immunofluorescence. 53BP1 (4937 Cell Signaling), BRCA1 (149823 Cell Signaling) and γH2AX (20E3 Cell Signaling) antibodies were used in immunofluorescence. Cells were imaged with a confocal microscope Leica AOBS SP8. Nuclei were stained with Hoechst 33342 (10 μg/ml, Merck). Unless specifically explained, images represent maximum intensity projections of 3D image stacks and were adjusted for brightness and contrast for optimal visualization.

Cell lysates after SDS-PAGE and immunoblotting on nitrocellulose (Whatman) were incubated with primary antibodies. HPR-conjugated secondary antibodies were obtained from cell signaling and blots were developed with Super Signal West Dura (Thermo Fisher Scientific). Odyssey Infrared Imaging systems (LI-COR Biosciences) was used to detect the fluorescence signal of secondary fluorescent antibodies (anti-Rabbit CF770 SAB4600215, anti-Mouse CF680 SAB4600361 Merck, Germany). For stripping, primary and secondary antibodies were removed by using Restore PLUS Western Blot Stripping Buffer (Thermo Fisher Scientific, USA), according to manufacturer. Unless otherwise indicated, all the immunoblot figures were representative of at least two biological replicates. The primary antibodies used in this work are listed in Supplementary Table S2.

### Dot blot for R-loop quantification

gDNA was extracted using the Quick-DNA Miniprep Kit (Zymo Research). The isolated gDNA was treated with 4 units (U) of Ambion™ RNase III (Thermo Fisher Scientific) for 20 min at 37°C. Samples were then split in half and control samples were digested with 10 U of RNase H (Thermo Fisher Scientific, USA) for 15 min at 37°C. Around 100–500 ng of the samples were spotted onto a nitrocellulose membrane (Amersham, UK) using a dot-blot apparatus (Thermo Fisher Scientific). DNA was cross-linked to the membrane by UV light, followed by blocking with 1x SSC buffer solution. The membrane was incubated overnight at 4°C with the S9.6 antibody (MABE1095 Merck, Germany). After incubation with HRP-conjugated secondary antibodies (Thermo Fisher Scientific, USA), the signal was detected using Super Signal West Dura (Thermo Fisher Scientific, USA). An antibody against dsDNA (MAB1293 Merck, Germany) was used as a loading control after stripping the membrane.

### RPA32 phosphorylation *in vitro*

HEK-293 cells were lysed in buffer A (25 mM Tris–HCl pH 8, 100 mM NaCl, 10% glycerol) by sonication. The lysate was clarified by centrifugation at 20 000 × g for 10 min. Protein concentration was determined using a Bradford assay and adjusted to 10 mg/mL. The following components were combined in buffer B (40 mM HEPES pH 7.5, 8 mM MgCl_2_, 0.5 mM DTT, 3 mM ATP): 293T extract (2-5 mg/mL final concentration) and 2 mM purified RPA (final concentration 2.16 µM). This reaction was incubated at 37°C for 2 h.

Phosphorylated RPA32 was purified using Glutathione Sepharose 4B resin. RPA phosphorylation was assessed by Western blot using the following antibodies: pRPA32 Ser 4/8 (A300-245A Bethyl, Montgomery, Texas, USA) and pRPA32 Ser 33 (A300-246A Bethyl, Montgomery, Texas, USA).

### Oligonucleotides, UV melting, and Thioflavin T fluorescence-based G4 detection

The oligonucleotides have been purchased from Microsynth-AG (Balgach, Switzerland), or alternatively from MWG Eurofins (Ebersberg, Germany). Their sequences are reported in Supplementary Table S1. DNA concentration was determined from the absorbance at 260 nm of the oligonucleotides diluted in milli Q water using as extinction coefficients 7500, 8500, 15 000, and 12,500 M–1 cm–1 for C, T, A, and G, respectively. The oligonucleotides were HPLC purified. UV melting was performed by using a Jasco V-750 UV–visible spectrophotometer equipped with a Peltier temperature control system (ETCS-761) (Jasco Europe, Cremella, Italy). The spectra were analyzed with Spectra Manager (Jasco Europe, Cremella, Italy). The oligonucleotides (1 μM) were annealed in 50 mM Na-cacodylate (pH 7.4) and 100 mM KCl (5 min at 95°C, overnight at RT). The melting curves were recorded at 295 nm in a 0.2 cm path length quartz cuvette, heating (25–95°C) at a rate of 0.5°C/min.

2-[4-(dimethylamino)phenyl]-3,6-dimethyl-1,3-benzothiazol-3-ium chloride (Thioflavin T, ThT, Sigma-Aldrich, Calbiochem) was prepared in DMSO at a concentration of 5 mM. Oligonucleotides and ThT were mixed at 1 and 0.5 µM final concentrations in 100 µl of final volume. Fluorescence emission was collected at 485 nm after excitation at 425 nm in a microplate reader (BioTek Synergy H1, Biotek, USA) and expressed as increase of fluorescence quantum yield at 485 nm in the presence of G4-forming sequences [50].

### Oligonucleotide-based R-loop and G-loop formation *in vitro*

To create synthetic R-loop and G-loop substrate, three oligonucleotides were annealed: the template DNA strand, the annealed RNA strand and the displaced non template DNA strand. For G-loop, the sequence of motif A or the 32R element of *KRAS* were introduced into the non-template DNA strand. To ensure the formation of a G4 structure rather than a canonical duplex, the template DNA strand was designed to exclude the G4 motif of non template DNA strand. RNA-DNA hybrids were created using a ratio of 1:2 RNA:DNA. Specifically, 800 pmol each of the bottom-strand DNA (DNA1_Rloop), 5′ Cy5.5-labeled RNA strand (RNA_Rloop) and top-strand DNA (DNA2_Rloop) were annealed in annealing buffer (50 mM Tris–HCl (pH 7.4) and 100 mM KCl) by heating to 98°C and then cooling slowly to 25°C over 2 h to generate a R-loop. DNA1_Rloop + RNA_Rloop + DNA2_MotifA_Rloop or DNA1_Rloop_G4_32R (these latter containing QPS) were similarly mixed to generate G-loop, while DNA1 and DNA2 were mixed to generate DNA:DNA molecules and DNA1 and RNA_Rloop were mixed to generate DNA:RNA heteroduplexes. The sequences of these oligos are provided as Supplementary Table S1. The mixtures were stored at 4°C for future use.

For the *in vitro* assay, indicated pmol of either the R-loop, G-loop or duplex were mixed with scaling amounts of RNase H1 (ENZ-164 ProSpec, Israel or home-made) and 2.16 µM of recombinant RPA complex (CUSABIO Technology LLC, USA or home-made). The reaction was conducted in RNase H1 reaction buffer 10X (200 mM Hepes, 500 mM KCl, 40 mM MgCl₂, 10 mM DTT) and incubated at 37°C for 20 min. The reaction mixtures were then loaded onto an 8% TBE (1X) polyacrylamide gel and run in TBE1X at 80V for 1 h at 16°C. After electrophoresis, the gel was analyzed with the Odyssey CLx Imaging System (Li-COR Biosciences, Lincoln, USA), stained with EtBr (0.5 µg/ml in distilled water) and digitally acquired using a GelDoc system (Bio-Rad, Hercules, CA, USA).

### RNase H1 ribonuclease activity assay

DNA:RNA hybrids containing RNA labeled at the 5′ end with Cy5.5 were incubated with increasing amounts of RNase H1 (ENZ-164 Prospec, Israel or home-made), in the presence or absence of the RPA complex, using 1X RNase H1 Reaction Buffer.

The ribonuclease activity was monitored using a BioTek Synergy microplate reader, configured to maintain the temperature at 37°C. Fluorescence readings were taken every 20 s for a total duration of 30 min (Ex: 675 nm; Em: 694 nm; Gain: 150).

### GST pull-down assay from cell lysates

HEK-293 cells were transfected with 9 µg of pEGFP-N1 RNase H1 and incubated for 48 h. Glutathione Sepharose 4B resin was washed in PBS (with protease inhibitors) for 10 min at 4°C and then incubated with 6 µg of GST-RPA70 mutants. GST-MEF2D was used as a negative control. The GST-protein samples were rotated at 4°C for 4 h. 1 µg of each protein sample was reserved as input and mixed with 2X Laemmli sample buffer. Transfected 293T cells were washed with PBS, lysed with lysis buffer (300 mM NaCl, 50 mM Tris–HCl pH 7.5, 0.5% NP-40, 10% glycerol) supplemented with protease inhibitors and sonicated for 5 min using a Bioruptor Plus (Diagenode, Seraing, Belgium) with 30-s pulses. After centrifugation, the supernatant was incubated with GST-tagged proteins. The samples were rotated at 4°C overnight. The next day, the samples were washed three times with PBS and the complex collected with Glutathione Sepharose beads (Cytiva, USA). Laemmli 2X sample buffer was added to the dry resin to elute bound proteins.

### Surface plasmon resonance for the determination of RNase H1 and GST-RPA binding affinity and rate constants

A BIACORE X100 system (Cytiva-Pall, Marlborough, MA, USA) was used to analyze molecular interactions using SPR. His-RNase H1 was expressed in BL21-DE3 bacteria, grown at 37°C up to reaching O.D. = 0.6 and induced with 0.5M IPTG o/n at 16°C. Ni-NTA Lysis Buffer (50 mM NaH_2_PO_4_, 300 mM NaCl, 1 mM imidazole, pH 8) was used to resuspend bacterial pellet (10 ml per liter) and incubated in ice for 30 min. Five sonication pulses (50% power) were applied to complete the lysis (Bandelin Sono Plus). Lysate was cleared by centrifugation and the soluble fraction was applied to Ni-NTA Sepharose packed into gravity flow column (2 ml Ni-NTA sepharose per liter of bacteria culture). After washings (50 mM NaH_2_PO_4_, 300 mM NaCl, 10 mM imidazole), His-RNase H1 was eluted with 250 mM imidazole and subjected to dialysis against 1 liter of DB buffer (30 mM HEPES pH 7.4, 0.15 M NaCl, 0.005% NP-40). As we failed to induce the expression of individual RPA70, we used the trimeric complex expression system previously developed [51]. RPA and RPA Asp mutant (RPA70, RPA32 Asp8, RPA14) complexes were produced in BL21 DE3 following a previously established protocol with some modifications [51]. Briefly, bacteria were grown in 0.5 liter LB at 37°C up to reaching O.D. = 0.6. RPA70, RPA32, RPA14 protein expression was induced with 0.25 M IPTG (Merck) at 24°C for 6 h. Bacteria were pelleted and resuspended in 5 ml lysis buffer LB1 (30 mM HEPES pH 7.4, 0.25 mM EDTA, 0.01% NP-40, 0.25% inositol). After centrifugation and elimination of insoluble fraction, the supernatant was applied to a 10-ml Affi-Gel blue column (Bio-Rad) equilibrated with LB2 (LB1 containing 50 mM KCl) and sequentially washed with LB3 (LB1 containing 800 mM KCl) and LB4 (LB1 containing 0.1 M sodium thiocyanate). RPA complex was eluted with LB5 (LB1 containing 1.5 M sodium thiocyanate) and dialysed against 1 liter of DB buffer (30 mM HEPES pH 7.4, 0.15 M NaCl, 0.005% NP-40). RPA and RPA Asp complexes were separately coupled to a carboxymethylated dextran surface of the CM5 sensor chip using amine-coupling chemistry. The ammine-coupling procedure was performed by setting the instrumentation temperature at 25°C using the running buffer HBS-P (0.01 M HEPES pH 7.4, 0.15 M NaCl, 0.005% v/v Surfactant P20) at a flow rate of 5 µL/min and with the following three steps as recommended by the producer (Biacore Sensor Surface handbook BR100571) and adapted for the purpose. First step, the CM5 chip was activated by injecting EDC/NHS (1/1) on both flowcells 1 and 2 for 10 min. Second step, RPA complexes were diluted in sodium acetate 10 mM pH 4.5 at final concentration of 0.075 µg/mL and injected on flow cell 2 until till reaching a surface density of 3577 RU. Third step, 1 M ethanolamine-HCl pH 8.5 was injected on both cells for 10 min. Appropriate, multiple concentrations of RNase H1 (7.3, 3.6, 1.8, 0.9, 0.45, 0.113, and 0.057 µM) were injected for 5 min at 25°C and flow rate of 10 μL/min in running buffer HBS-P+ (0.01 M HEPES pH 7.4, 0.15 M NaCl, 0.05% v/v Surfactant P20). After injection, analyte solutions were replaced by running buffer at a continuous flow rate of 10 μL/min. Surface regeneration was accomplished by injecting 100 and 250 mM NaOH for a contact time of 1 min. Each sensorgram was subtracted for the response observed in the control flow cell (no immobilized protein) and normalized to a baseline of 0 RU. The sensorgram curves were acquired by setting the BiacoreX100 Control software, 2.0.2 (Cytiva-Pall, Marlborough [HQ], MA, USA) in manual run. SPR Biacore run was performed at Cytiva center at Università Statale di Milano (Milan, Italy).

### Fluorescence polarization binding assay

Fluorescence polarization (FP) values were calculated using equation (1), where *S* is the fluorescence intensity of emitted light parallel to the excitation beam, *P* is the fluorescence intensity of emitted light perpendicular to the excitation beam, and *G* is the correction factor accounting for instrument bias.


(1) \begin{eqnarray*}
FP = 1000 \times \frac{{(S - GP)}}{{(S + GP)}}
\end{eqnarray*}

The *G* factor was experimentally determined using the fluorescent probe alone.

Protein deadRNase H1 was diluted in 20 mM HEPES (pH 7.0), 100 mM NaCl, and 1 mM DTT to final concentrations ranging from 0.03 to 30 μM. Titration assays were performed using fluorescently labeled probes (FITC–G-loop, FITC-R-loop heteroduplex, and RNA) at a final concentration of 30 nM. Each mixture (100 μL) was transferred into black 96-well OptiPlate microplates (PerkinElmer, Buckinghamshire, UK) and incubated at room temperature for 30 min. Fluorescence polarization signals were recorded at 25°C using a VICTOR Nivo Multimode Microplate Reader (Revvity, PerkinElmer, Buckinghamshire, UK) equipped with a 480 nm excitation filter, a 535 nm emission filter, and a 505 nm dichroic mirror. Orbital shaking was applied (200 rpm for 0.1 s). The average FP values from at least three independent experiments were plotted as a function of deadRNase H1 concentration. Equilibrium dissociation constants (*K*_D_) were obtained by nonlinear regression analysis of polarization (FP) versus total protein concentration (*P*_T_) using equation (2):


(2) \begin{eqnarray*}
F &=& {F}_{L} + ({F}_{LP} - {F}_{L})\frac{1}{2L_{T}}\left[({L}_{T} + {P}_{T} + {K}_{D})\right. \\&&\left.- \,\sqrt {{({L}_{T} + {P}_{T} + {K}_{D})}^{2} - 4{L}_{T}{P}_{T}} \right]
\end{eqnarray*}

where *F* is the measured mean fluorescence polarization, *F*_L_ is the polarization of the free labeled ligand, *F*_LP_ is the maximum polarization of the ligand–protein complex, and *L*_T_ is the total concentration of labeled ligand.

### Mass spectrometry-based immuno-precipitation proteomics

Around 5 × 10^6^ BJ/hTERT HRAS^G12V^-ER cells, induced or not with 4-OHT to express HRAS, were harvested and lysed with 2 ml of the hypotonic buffer described above. 400 μg lysates were incubated O/N with 16ul Dynabeads Protein G pre-bound to 3 μg anti-RPA70 antibody (Millipore, RRID: AB_565121) or mCherry (Thermo Fisher Scientific, RRID: AB_3666174) as a control. After four washes with lysis buffer, the immunocomplexes were reversed with 2 × Laemmli sample buffer and resolved by SDS-PAGE. After in gel trypsin digestion, peptides were resuspended in 20 μl of 0.1% formic acid and then subjected to Nano UPLC (Ultimate 3000 nano UHPLC system, ThermoFisher Scientific, USA) MS/MS analysis. The full scan was performed between 300 and 1650 m/z at the resolution 60 000 at 200 m/z, the automatic gain control target for the full scan was set to 3e6. The MS/MS scan was operated in Top 20 mode using these settings: resolution 15 000 at 200 m/z; automatic gain control target 1e5; maximum injection time 19ms; normalized collision energy at 28%; isolation window of 1.4 Th; charge sate exclusion: unassigned, 1, >6; dynamic exclusion 30 s. Raw MS files were analyzed and searched against Homo sapiens protein database based on the species of the samples using Maxquant (1.6.2.6). The parameters were set as follows: the protein modifications were carbamidomethylation (C) (fixed), oxidation (M) (variable); the enzyme specificity was set to trypsin; the maximum missed cleavages were set to 2; the precursor ion mass tolerance was set to 10 ppm, and MS/MS tolerance was 0.5 Da. MS/MS analysis was performed by Creative Proteomics (Shirley, USA). For the functional enrichment analysis (shown in Supplementary Fig. S5B) of the 48 RPA70 interactors identified as significantly associated with RPA70 in proliferating BJ-hTERT cells, but not, or to a significantly lesser extent, in BJ-hTERT/HRASG12V cells, the hits were selected based on differential interaction analysis: Log2(foldchange + RAS vs -RAS) > 0.5;^∗^*P*^∗^<0.1 from a total of 167 RPA70 interactors. Functional enrichment analysis was performed using g:Profiler (RRID: SCR_006809), focusing on Gene Ontology biological processes and pathway annotations. Enriched functional categories were ranked according to fold enrichment.

### Chromatin immunoprecipitation, library construction, ChIP-seq, DRIP-seq, and NGS data analysis

Chromatin was extracted from BJ/hTERT, BJ/hTERT/HRAS^G12V^-ER, BJ-hTERT/HRAS^G12V^-ER/HDAC4-FLAG, BJ-hTERT/HRAS^G12V^-ER/RPA70-GFP and BJ-hTERT/HRAS^G12V^-ER/H2B-GFP cells and immunoprecipitated with 2 μg of BRCA1 (149823 Cell Signaling Technology), 3 μg of IgG (Active Motif), 4μg of RNA Pol II Ser5 (1H4B6 Merck), 3 μg of RPA32 (2208 Cell Signaling Technology), 3 μg of phosphoRPA32 (A300-245A Bethyl), 3 μg of RNase H1 (Merck), and γH2AX (9718, Cell Signaling Technology) antibodies.

For each ChIP, approximately 3 × 10^6^ BJ cells were used. Cells were treated as indicated and DNA-protein complexes were cross-linked with 1% formaldehyde (Sigma-Aldrich) for 15 min at room temperature. ChIP was performed as previously described [13]. DNA SMART ChIP-Seq Kit (Takara) was used for library generation of samples subjected to Illumina sequencing. To generate libraries with the DNA SMART ChIP-Seq Kit, a priming site was added to the 3′ end of the DNA template using the Terminal Deoxynucleotidyl Transferase. This was followed by annealing of a Poly(dA) Primer, which anneals to the T-tail. This primer was then used by the SMARTScribe RT to copy the DNA strand. Sequencing libraries were then generated by PCR-mediated addition of Illumina adapters and sequenced on Illumina NovaSeq 6000 (PE150, Genewiz, Germany). DNA nanoball (DNB) technology was applied for library preparation of samples subjected to BGI-seq 500 sequencing (SE50, BGI, China) following manifacturer (Roche, Switzerland).

The DRIP-seq protocol was optimized for 8 × 10^6^ cells. Total nucleic acids were extracted from indicated BJ clones by SDS/Proteinase K treatment at 37°C followed by phenol-chloroform extraction and ethanol precipitation. DNA was restricted with a cocktail of 30U each of five enzymes (HindIII, EcoRI, BsrGI, XbaI, and SspI, NEB, USA) for 6 h. 10 µg of restricted gDNA was digested or not with 10 U RNase H (NEB) for 2 h at 37°C, brought to 450 μl with milliQ water and incubated O/N at 4°C by adding 50 μl of 10X binding buffer (100 mM NaPO4 pH 7, 1.4 M NaCl, 0.5% Triton X-100) with 10 μg of S9.6 antibody (MABE1095, Merck, Germany) or IgG as a control (Abcam, UK). 8 μl magnetic Dynabeads protein G (Thermo Fisher Scientific, USA) were used to collect antibodies (2h, at 4°C). After three washes in 1X binding buffer, beads were incubated in 250 μl of elution buffer (50 mM Tris-Cl pH 8, 10 mM EDTA pH8, 0.5% SDS, 140 μg Proteinase K) with rotation at 55°C for 45 min. After DNA extraction, the pulled-down DNA (with the negative control of RNase H-treatment) and input DNA were sonicated for 5′ (30′′ON/OFF cycles, Bioruptor, Diagenode) and size-selected with Agencourt (MA, USA) beads. Sequencing libraries were then generated by PCR-mediated addition of Illumina adapters and sequenced on Illumina HiSeq 4000 (PE150, Genewiz, Germany).

The antibodies used in ChIP-seq and DRIP-seq experiments were validated using appropriate controls. Antibody validation is provided in Supplementary Fig. S1A–H.

For ChIP-seq and DRIP-seq data analysis, the FastQC/MultiQC programs were used to evaluate the quality of sequencing reads and Bowtie 2 or bwa-mem were used to align them to NCBI GRCh38 human genome reference. Peak calling was performed against input sequences using EDD for γH2AX ChIP (–write-log-ratios and –write-bin-scores options) and MACS3 for other ChIP and DRIP samples (–nomodel, according extsize and -q 0.05 or -p 0.05 options); IDR peaks identified by comparing the replicates were considered for analysis, as previously described [20]. Gene annotations were performed as previously described [13]. gplots, biomaRt, ggplot2, and Gviz R/Bioconductor packages and the deepTools suite were used to generate peak heatmaps, metaplot and profiles, as previously reported [13]. WindowBed 2.31.1 was used to identify a minimal overlap of at least 1 nucleotide between investigated markers. UCSC Wiggle (Wig) Track Files v1.5.4 were created after peak signals conversion in normalized read counts (1 Mb window). BigWig files used in deepTools were RPKM-normalized (Reads Per Kilobase per Million mapped reads), accounting for both sequencing depth and feature length. Coverage was calculated using a binsize of 50 bp, and normalization was performed to ensure comparability across samples. bigwigCompare tool was used to compute pRPA32 rate (pseudocount:11, binsize:50). multiBigwigSummary tool was used to average scores for every genomic region for every bigWig file (binSize: 10000). PCA and Spearman correlation were computed by using multiBigwigSummary file (binSize: 10000, ntop: 1000). Genomic loci of interest and ChIP-seq signal traces were visualized using the Integrative Genomics Viewer (IGV, RRID:SCR_011793) to facilitate inspection of peak profiles and chromatin features. Motif discovery was performed using the MEME Suite 4.11.4 (RRID:SCR_001783), specifically employing the MEME algorithm in ZOOPS mode (Zero or One Occurrence Per Sequence), with motif lengths set between 6 and 50 nt. Prediction of G4-forming sequences was carried out using the QGRS Mapper (https://bioinformatics.ramapo.edu/QGRS/index.php) with the following parameters: maximum length = 30, minimum G-group size = 2, loop size = 0–36. G4Hunter (RRID:SCR_018964) was queried using the motifs identified by CentriMO to detect consensus sequences of putative quadruplex-forming sequences (QPS), applying a word size of 25 and a threshold score of 1.2. To identify putative G-loop structures, DNA:RNA hybrids enriched peaks identified by DRIP-seq were oriented according to the direction of transcription, and BG4-seq enriched peaks were mapped to either the template or non-template DNA strand in relation to the presence of QPS.

For differential quantitative analysis of DRIP signals, MACS2 bdgdiff (version 2.2.9.1+) was used to compare bedGraph files in the BJ + RAS condition relative to BJ + RAS/HDAC4. The following parameters were applied: –cutoff 0.5: minimum fold enrichment required to call a differential region; –min-len 200: minimum length of differential regions; –depth1 14 and –depth2 14: sequencing depth normalization for each condition; –max-gap 100: maximum gap allowed between enriched regions to be merged. MACS2 bdgdiff was used as it outperforms DiffBind when peaks are broad.

TrAEL-seq reads (GEO: GSE299123) from 5′-trimmed BAM files were quantified in 50 kb bins and the day 3/day 0 ratio after RAS induction in IMR90 was calculated to generate bedGraph and bigWig tracks. These were processed using computeMatrix (bin size = 20) to produce the metaplot in Fig. 8G. BAM files were also used to quantify read density around predefined R-loop regions for Fig. 8M.

For differential quantitative analysis of RNase H1 ChIP-seq signals, DiffBind 3.18.0 was employed to analyze bigWig files and identify genomic regions selectively bound by RNase H1 in either the H2B + RAS or RPA70 + RAS conditions. Normalization was performed using DESeq2 (default method), adjusting for library size and composition bias. Statistically significant differences in RNase H1 binding were identified using adjusted p-values < 0.05 and |log₂(Fold-change)| > 1 as fold-change thresholds. The workflows and bioinformatic analyses performed for this study have been uploaded to the Galaxy platform [52] and made available for consultation at the following link: https://usegalaxy.org.au/u/eros87/h/rip111224

### Molecular docking

The crystal structure PDB: 3bsu [53] was employed for the human RNase H1 hybrid-binding domain (residues 27–74), 3bsu was the only experimental structure available in the PDB database when this manuscript was submitted. For the RPA70 OB domain (residues 1–120), three different resolved structures were selected: the crystal structure of (i) the human RPA70 OB domain, in its apo form (PDB: 2b29 [54]), (ii) the human RPA70 *N*-terminal domain bound to MRE11 (PDB: 8k00 [55]), and (iii) the human RPA70 *N*-terminal domain bound to the ligand 2P9 (PDB: 4o0a [56]). Both holo forms in (ii) and (iii) correspond to the same RPA70 binding site as that experimentally observed with RNase H1 interaction (see Results section).

HADDOCK and ClusPro protein-protein docking web interfaces were employed for performing docking of the selected crystal structures. RPA70 and RNase H1 binding sites were defined by selecting those amino acid residues belonging to the regions confirmed to participate in the binding and whose side chains were exposed to the surface. Thus, residues 33, 41, 43, 57, 85, 87, 91, 93, and 95 from the RPA70 OB domain and residues 63–73 from the RNase H1 hybrid-binding domain were selected as “active residues” in HADDOCK and as residues with “attraction” in ClusPro. Standard parameters (by default) were used with HADDOCK and ClusPro webserver. Overall, six docking runs were performed by combining each of the selected crystal structures.

For each docking run, the top two RPA70:RNase H1 complexes, as ranked by each docking algorithm were selected (overall, 12 docked structures). Their binding conformations were grouped in three different binding modes, or poses, according to the orientation of RNase H1 with respect to the RPA70 binding site.

### Molecular dynamics (MD)

Each RPA70:RNase H1 complex was first minimized without solvent by using the GROMACS package (RRID: SCR_014565). AMBER99SB-ILDN [57] force field was employed for the parameterization of the system. Each minimized structure was then immersed in a cubic box of TIP3P water and neutralized by adding Na+/Cl- counterions. The solvated system was subjected to a second minimization, followed by 200 ps NVT and 500 ps NPT equilibrations at 200 K, in which the geometry of the protein complex was restrained to ensure its integrity. Each NPT MD production simulation started with an annealing phase to rise the temperature linearly from 200 to 300 K during the first 100 ns, followed by an additional 400 ns simulation at constant temperature (300 K). The combined simulated time was 6 μs (12 complexes each for 500 ns). Periodic boundary conditions, Verlet integrator and LINCS constraints were used with a time step of 2 fs. The temperature was controlled utilizing a modified Berendsen thermostat, while an isotropic pressure of 1 bar was kept using the Parinello-Rahman barostat. A cutoff of 0.9 nm was used for the electrostatic and van der Waals non-bonded interactions. The analysis of the resulting MD production trajectory (stripped of solvent and counterions) was conducted as implemented in the VMD software package (RRID: SCR_001820) and GROMACS (RRID: SCR_014565).

### MD protocol when including disordered regions to RNase H1 hybrid binding domain

The three binding poses identified through docking calculations were refined by grafting the disordered regions of RNase H1 into the structure. In particular, residue fragments 18–26 and 75–82 were added to the extremes of the RNase H1 hybrid-binding domain structure. The new complexes were prepared, minimized, and equilibrated as detailed above. A structural sampling phase followed: before the MD production phase, a 300 ns MD simulation of the RPA70-RNase H1 complex at 400 K was performed to obtain a structural sampling set of the disordered regions. The backbone of the structured regions were restrained in the simulation to preserve the binding conformation while the unstructured regions were free to explore all the accessible binding conformations. A cluster analysis of the set of structures (represented only as alpha-carbons) from the trajectory was performed by employing the Daura’s algorithm. The RMSD cut-off for two structures to be neighbor was set to 0.2 nm. The most representative conformation of the biggest cluster was selected for the subsequent MD production phase, since this should correspond to the binding conformation with the highest population in the MD trajectory. After the MD production phase, which was performed as explained above, an additional 500 ns MD simulation at 330 K was performed to ensure the stability of each binding pose.

### Statistical analysis

For experimental data, Student t-test was employed. Mann–Whitney test was applied when normality could not be assumed. *P* < 0.05 was chosen as statistical limit of significance. For comparisons between more than two samples, Anova test was applied coupled to Kruskal–Wallis and Dunn’s Multiple Comparison Test. For binary matched-pairs data, pairwise t-test was used. Excel Statistics (RRID: SCR_017294) and GraphPad Prism (RRID: SCR_002798) were used for routine analysis. We marked with ^∗^  *P* < 0.05, ^∗∗^  *P* < 0.01, ^∗∗∗^  *P* < 0.001. Unless otherwise indicated, all the data in the figures were represented as arithmetic means ± the standard deviations from at least three independent experiments.

## Results

### RAS-induced senescence is characterized by the accumulation of irreparable DNA damage, DNA:RNA hybrids and unsuccessful loading of BRCA1

**Figure 1.**
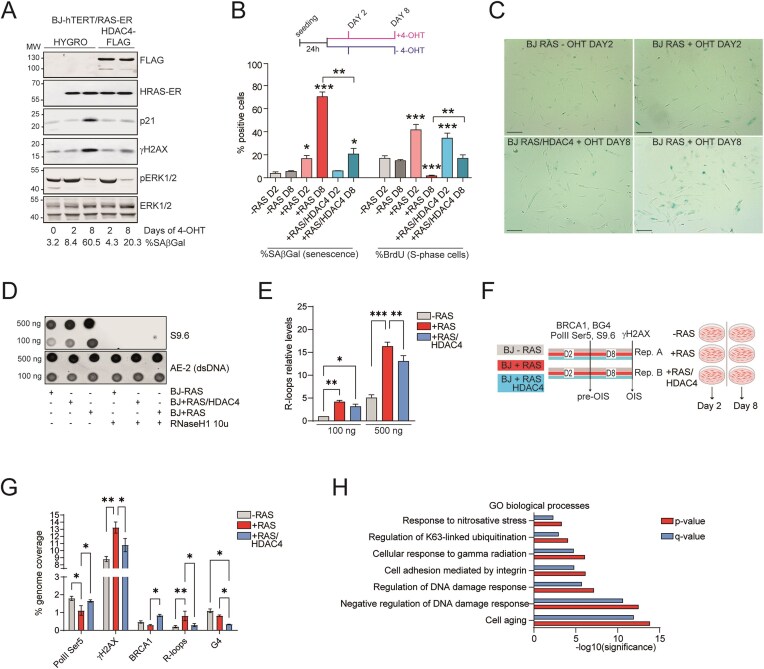
RAS-induced senescence is characterized by the accumulation of irreparable DNA damage, DNA:RNA hybrids and unsuccessful loading of BRCA1. A. Immunoblot analysis of the indicated proteins in BJ-hTERT/RAS-ER cells, expressing the indicated transgenes and treated or not as indicated with 4-OHT (1µM). % of SA-β-gal positivity is indicated. B. Quantification of SA-β-gal- positive cells and S-phase entering cells (BrdU assay, BrdU pulse of 3 h) in BJ-hTERT/RAS-ER Hygro cells or BJ-hTERT/RAS-ER HDAC4 cells, treated (+RAS) or not (-RAS) with 4-OHT for the indicated days (D2 = day2, D8 = day8) to induce HRAS^G12V^ expression. Mean ± SD; *n* = 4. *P < 0.05, **P < 0.01 and ***P < 0.001 (Dunn’s Multiple Comparison Test with respect to -RAS D2). Pairwise t-test was applied to indicated comparisons. C. Representative microscopic images of SA-β-gal stained BJ-hTERT/RAS-ER Hygro cells or BJ-hTERT/RAS-ER HDAC4 cells at the indicated time (days) after 4OHT treatment (Bar = 40 μm). D. Dot blot analysis using S9.6 antibody to detect R-loops and AE-2 antibody to detect dsDNA. Around 100 and 500 ng of nucleic acids extracted from the indicated cells grown for 2 days in presence or absence of 4-OHT were treated or not with 10u of RNase H and spotted on nitrocellulose film. E. Quantification of Dot blot shown in D. Mean ± SD; *n* = 3. F. Schematic representation of the ChIP-seq and DRIP-seq experiments performed in the indicated cells at the indicated time after 4-OHT treatment. G. Histogram representing the percentage of genome coverage of RNA PolII Ser5, γH2AX, BRCA1, R-loops and G4s enriched peaks in the indicated cells. Mean ± SD; two independent sequencing each coming from three biological replicates. *P < 0.05, **P < 0.01 and ***P < 0.001. (Dunn’s Multiple Comparison Test with respect to -RAS D2). Pairwise t-test was applied to indicated comparisons. H. *P*-value and q-value of pathway enrichment analysis performed by using MSigDB (RRID:SCR_016863) on genes associated to DNA:RNA hybrids found exclusive of BJ + RAS condition.

Induction of HRAS^G12V^ expression (HRAS-ER induced with 4-OHT) in BJ-hTERT cells resulted in premature onset of senescence within 8 days, named OIS or RIS [13]. RIS is characterized by increased levels of γH2AX, activation of CDK inhibitors (p21) and feedback repression of the MAPK pathway (dephosphorylation of ERK1/2) (Fig. 1A-C). Entry into the OIS was accompanied by increased proteasomal degradation of HDAC4 [13]. Notably, HDAC4 overexpression in BJ-hTERT/HRAS cells allowed bypass of RIS [13] (as shown by decreased positivity to SA-βgal and increased entry into the S phase, Fig. 1B, C) which was characterized by the lack of accumulation of DSBs and p21, but not by the relief of ERK1/2 phosphorylation (Fig. 1A). It has been previously reported that oncogene-induced replication stress leads to the accumulation of DNA:RNA hybrids [28, 47]. The dot-blot analysis, in which the presence of DNA:RNA hybrids was tested using the S9.6 antibody after RNase III treatment to avoid dsRNA cross-reactivity, confirmed the accumulation of DNA:RNA hybrids in the RIS condition and their reduction in RIS-escaped cells (RAS + HDAC4) (Fig. 1D, E).

With the aim of determining the relationship between R-loop accumulation and DNA damage–associated genomic fragility leading to cellular senescence, we mapped R-loops genome-wide in BJ-hTERT, BJ-hTERT/HRAS (hereafter referred to as “pre-RIS”, at day 2 of RAS induction, or “RIS”, at day 8 of RAS induction) and BJ-hTERT/HRAS/HDAC4 (hereafter referred to as “RIS escape”) cells using DRIP-seq (with S9.6 antibody [58]) and DNA G-quadruplexes by G4 ChIP-seq (with BG4 antibody [59]). Additionally, we mapped transcription initiation sites (with RNApolII Ser5), HDR-repairable sites (with BRCA1) on day 2 after *HRAS* induction, and persistent γH2AX on day 8 after *HRAS* induction (Fig. 1F). The time frame was selected to capture the earliest phases of R-loops accumulation and the mechanisms regulating their resolution, in line with previous reports showing that R-loops accumulate within the first 72 h following HRAS expression [28], preceding replication fork slowing, which becomes evident from the second day of HRAS induction [8, 20, 28]. This integrative approach aimed to map R-loops and G-quadruplex (G4)-enriched regions and examine their correlation with BRCA1-associated sites and RNA Polymerase II Ser5 phosphorylation during the pre-senescent (herein defined as “pre-RIS”) phase, when RAS-expressing cells remain proliferative (at day 2 of HRAS induction). Additionally, we aimed to intersect these data with genomic regions exhibiting persistent γH2AX phosphorylation (at day 8 of HRAS induction).

The distribution of sequencing peaks relative to transcription start sites (TSS) and transcription end sites (TES) revealed patterns for DNA:RNA hybrids and R-loops identified by DRIP-seq, as well as G-quadruplexes mapped by G4-seq, which matched those reported in similar studies [60–64]. These structures showed enrichment at TSS, TES, and promoter regions (Supplementary Fig. S2A, S2B). RNA Polymerase II phosphorylated at Ser5 showed the expected sharp accumulation at the TSS, while BRCA1 exhibited a more heterogeneous distribution with a prominent peak overlapping the TSS (Supplementary Fig. S2A, S2B). Interestingly, the pre-RIS and RIS-escape conditions could be clearly distinguished in PCA plots based on the genomic localization of R-loops, G4s and BRCA1 (Supplementary Fig. S2C, S2D). Overall, the genomic distribution of γH2AX was comparable across the three cellular models, with increased signal levels in the RIS condition, especially within coding exons and introns (Supplementary Fig. S2E). Fingerprint analysis revealed a more uniform genome-wide distribution of γH2AX signal in RAS-expressing cells compared with normally proliferating BJ cells, consistent with broad γH2AX accumulation in RIS. RAS + HDAC4-expressing cells displayed an intermediate profile, indicating partial restoration toward a more focal γH2AX distribution (Supplementary Fig. S2F). Moreover, as controls, sequencing libraries were also attempted from chromatin immunoprecipitated with anti-FLAG antibodies (negative control for ChIP-seq) and from DRIP samples treated with bacterial RNase H (negative control for DRIP-seq). In both cases, the amount of recovered genomic material was insufficient to generate sequencing libraries, thereby confirming a favorable signal-to-noise ratio in the primary experimental conditions. Overall, HRAS-expressing cells exhibited broader genomic regions marked by γH2AX, an increased prevalence of R-loops, a modest reduction in G-quadruplex abundance, and a reduced capacity to recruit BRCA1 to chromatin compared with normally proliferating BJ cells (Fig. 1G). Senescence-escaping cells overexpressing HDAC4 and HRAS displayed a profile largely similar to that of normally proliferating BJ cells (Fig. 1G), with the exception of G-quadruplexes, which were further reduced relative to both normal proliferating BJ cells and pre-RIS cells (Fig. 1G). This reduction may be attributable to partial heterochromatinization induced by ectopic HDAC4 expression, as previously reported [20, 65, 66].

Although the distribution of DRIP-seq enriched peak signals was similar across the three cellular conditions analyzed (normal growing, pre-RIS, and RIS-escapers), with preferential accumulation at TSS and TES (Supplementary Fig. S2A, B), DNA:RNA hybrids were found to be significantly more abundant in pre-RIS cells (Fig. 1G). This likely reflects transcriptional resetting driven by HRAS expression and the activation of a senescence-associated transcriptional program. To validate this observation, we isolated R-loops specifically enriched in pre-RIS cells compared with normal growing BJ cells and identified the associated genes by using GREAT 4.0 [67] (Supplementary Table S3). Pathway enrichment revealed signatures characteristic of cellular senescence, DNA damage response activation, and metabolic adaptations typical of the senescent state (Fig. 1H) [13]. Based on these findings, subsequent analyses focused exclusively on differential R-loops between pre-RIS (BJ + RAS) and RIS-escape condition (BJ + RAS/HDAC4).

In summary, this analysis indicates that early stages of senescence, compared to normal proliferation and RIS escape, are characterized by R-loops accumulation and reduced chromatin binding capacity of BRCA1, which correlate at a later phase with an expansion of the regions marked by γH2AX.

### The co-occurrence of R-loops and G-quadruplex structures characterized the pre-RIS condition

To identify the repertoire of R-loops specific to the pre-RIS condition, we employed the MACS2 bdgdiff tool [68] to compare the four DRIP-seq bedgraph files from pre-RIS cells (BJ + RAS) and RIS-escaper cells (BJ + RAS/HDAC4). We applied a log₁₀ likelihood ratio cutoff of 0.5 (corresponding to a likelihood ratio of 500) and set a minimum DNA:RNA hybrid length of 50 bp to exclude short hybrids and priming sites.

**Figure 2.**
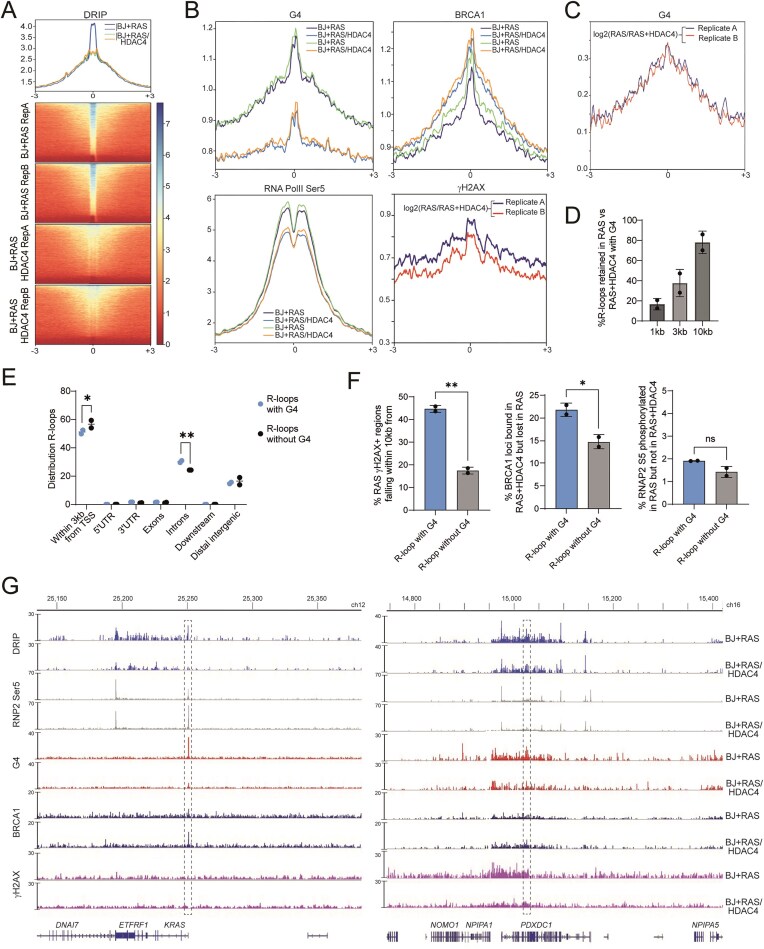
The co-occurrence of R-loops and G-quadruplex structures characterized the RIS condition. (**A**) Metaplot and heatmap of DRIP-seq signal 6kb around the 11 439 genomic regions with significant enrichment of DNA:RNA hybrids in BJ + RAS cells compared with BJ + RAS/HDAC4 cells. (**B**) Metaplot of RNA PolII Ser5, γH2AX, BRCA1 and G4 ChIP-seq signals within 6 kb around the 11 439 R-loops characterizing the pre-RIS condition. (**C**) Metaplot of log2 ratio of γH2AX signal in BJ + RAS with respect to BJ + RAS/HDAC4 cells within 6 kb around the 11 439 R-loops characterizing the RIS condition. (**D**) Histogram reporting the percentage of RIS-specific R-loops co-localizing with G4s within the indicated genomic interval. (**E**) Histogram reporting the genomic distribution of R-loops and “G-loop like” structures (defined as containing a G4 within 3 kb from DNA:RNA hybrids detected in DRIP-seq). (**F**) Histograms reporting the percentage of RIS-specific R-loops with G4 (defined as containing a G4 within 3kb from DNA:RNA hybrids detected in DRIP-seq) or without G4 co-localizing with G4s withint the indicated genomic interval in the indicated cellular conditions. (**G**) Distribution of R-loops, RNA PolII Ser5 (RNP2 Ser5), G4s, γH2AX, and BRCA1 on chromosome 12 and 16 in the indicated cells expressing or not RAS and HDAC4, as indicated. The highlighted region represents the genomic locus where a significant enrichment of the DRIP-seq signal is detected in BJ + RAS cells. At the *PDXDC1* locus, enrichment encompasses multiple distinct regions within the highlighted interval, which spans approximately 15 kb. In B and F, data are expressed as mean ± SD; two independent sequencing each coming from 3 biological replicates. *P < 0.05, **P < 0.01. Pairwise t-test was applied to indicated comparisons.

The analysis identified 11 439 genomic regions with significant enrichment of DNA:RNA hybrids and R-loops in pre-RIS cells compared with RIS-escaper cells (Supplementary Table S4). Consistently, the DRIP-seq signal profile confirmed a clear enrichment of R-loop signal in BJ + RAS cells (pre-RIS condition) relative to BJ + RAS/HDAC4 cells (RIS-escaper cells) (Fig. 2A). To assess the quality of our DRIP-seq data, we compared the pre-RIS-specific DRIP-seq peaks with those previously identified in an independent study using IMR90 human fibroblasts [69]. We observed a strong enrichment of DRIP-seq signal in both BJ + RAS and BJ + RAS/HDAC4 samples near the peaks reported in etoposide-treated IMR90 cells treated with etoposide in the earlier study (Supplementary Fig. S3A). Notably, a relevant proportion of the DRIP-seq peaks identified under pre-RIS conditions overlapped with those found in etoposide-treated IMR90 fibroblasts, suggesting the existence of a conserved core set of DRIP-seq peaks associated with senescence and replication stress (Supplementary Fig. S3B).

Next, we performed co-localization analysis within a ± 3 kb window around the pre-RIS-enriched R-loop peaks, integrating signals for γH2AX, BRCA1, RNA Polymerase II Ser5, and G4s across both conditions. This revealed a moderate increase in γH2AX and RNAPol2 Ser5 signals in pre-RIS cells, supporting the notion that R-loops accumulate at genomic regions characterized by γH2AX deposition and active transcription (Fig. 2B). Interestingly, a slight reduction in BRCA1 signal was observed at these loci in BJ + RAS cells, suggesting that R-loop accumulation in the pre-RIS state may interfere with BRCA1 recruitment.

Strikingly, G-quadruplex signal in RIS-escaping cells was significantly reduced compared with pre-RIS cells at these R-loop-enriched regions (Fig. 2C). Co-localization between R-loops and G4 structures has been reported in various models and cellular states [43, 70, 71]; here, we observed that 80% of pre-RIS-enriched R-loops co-localized with G4s within a 10 kb genomic window (Fig. 2D). Heatmaps of signal intensity within a 3 kb window centered on pre-RIS-enriched R-loop peaks confirmed the differences observed in the signal profiles of γH2AX, BRCA1, RNA Polymerase II Ser5 and G4s between the experimental conditions (Supplementary Fig. S3C). Pre-RIS-specific R-loops were predominantly located near TSS and within intronic regions (Fig. 2E). The presence or absence of G4 structures only slightly affected their distribution, with G4-associated R-loops being less proximal to TSSs and more intronic (Fig. 2E). It is noteworthy that R-loops co-occurring with G4 structures (G-loop-like) in BJ + RAS cells showed a tendency to accumulate within γH2AX-positive regions, which were also characterized by reduced BRCA1 binding (Fig. 2F).

Fig. 2G illustrates two representative genomic loci used to assess the quality of the immunoprecipitations, showing clear R-loop enrichment in BJ + RAS cells compared with BJ + RAS/HDAC4 cells. At the *KRAS* locus, a BJ + RAS-specific R-loop overlapped the TSS, in proximity to well-characterized G-quadruplex structures located within the proximal promoter region (classified as near, mid and far [72]). BRCA1 enrichment at this locus was observed exclusively in RIS-escaper cells (BJ + RAS + HDAC4), whereas no γH2AX enrichment was detected under either condition. Conversely, at the *PDXDC1* locus, an intronic R-loop specific to BJ + RAS cells was detected, associated with a strong G4 signal. BRCA1 showed no enrichment in both conditions, while γH2AX accumulation was observed in BJ + RAS cells. Validation of the G4-seq and DRIP-seq data by qPCR is shown in Supplementary Fig. S3D.

Collectively, these findings highlight a strong association between R-loop accumulation and G4 structures in pre-RIS cells, suggesting that transcriptional and structural features of chromatin in the pre-senescent state may affect DNA repair dynamics.

### G-loops exhibit resistance to RNase H1-mediated cleavage, suggesting a structural configuration that impairs enzymatic accessibility

G-loops are complex nucleic acid structures composed of a DNA G-quadruplex structure (G4) formed on the non-template DNA strand and an R-loop [43, 73, 74]. Although long hypothesized, their existence was only recently demonstrated in a seminal Science paper using plasmid DNA and nucleoplasmic extracts from Xenopus laevis [45]. According to their findings, trans-invasion of a G4 by an RNA molecule complementary to the template DNA strand leads to the formation of a G-loop. This structure is required for the recruitment of homologous recombination repair factors, helicases, and endonucleases, which sequentially resolve the G4 and excise the template DNA strand, ultimately completing DNA repair. In this model, co-occurring R-loops assist in G4 resolution. Conversely, failure to resolve G-loops results in their persistence, which is detrimental and contributes to genomic instability [45].

The mechanisms governing DNA:RNA hybrids processing and the extent to which delays in their resolution affect G-loop dynamics and genomic stability remain unclear. We hypothesized that impaired G-loop processing contributes to cell-cycle arrest in senescent cells. To address this, we proceeded as follows: (i) identification of *bona fide* G4 structures from our G4-ChIP-seq datasets; (ii) prediction of putative G-loops based on the co-occurrence of G4s on the non-template DNA strand and R-loops; (iii) assessment of the stability of these structures.

**Figure 3.**
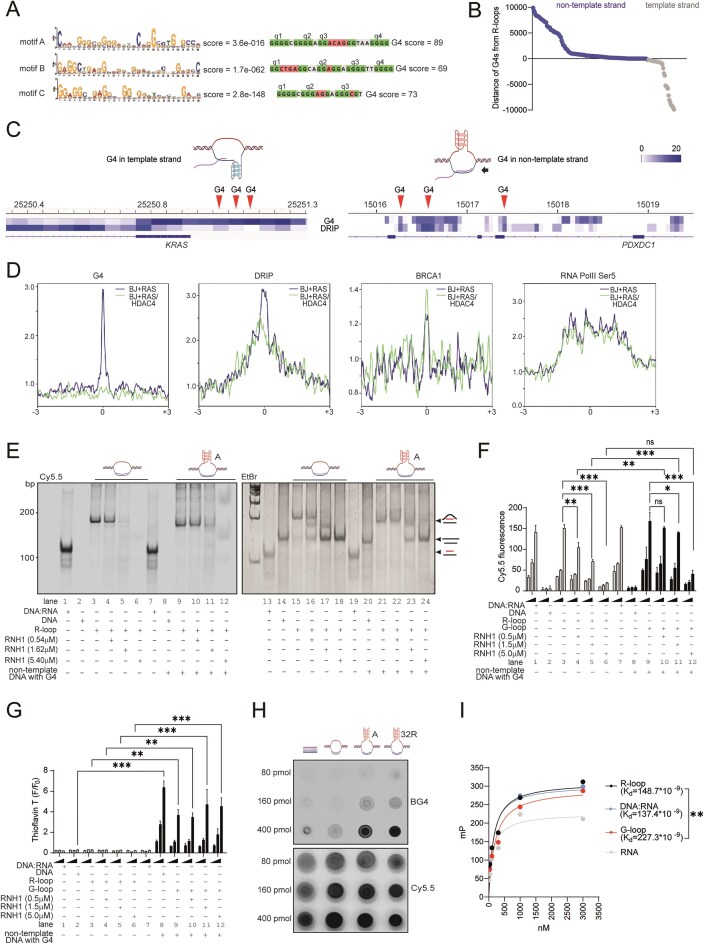
G-loops exhibit resistance to RNase H1-mediated cleavage, suggesting a structural configuration that impairs enzymatic accessibility. (**A**) Motif discovery analysis reporting the three most frequent motifs found enriched in G4 ChIP-seq in BJ + RAS cells and co-localizing with R-loops within 3 kb. Each motif was analyzed using G4Hunter to identify the optimal sequence containing a putative QPS. The G4Hunter score is reported along with the predicted tetrads. (**B**) Plot reporting the distances in BJ + RAS of the G4s (corresponding to the three motifs found in Fig. 3A) with respect to the closest R-loop, divided according to their co-occurrence on either the template or non-template strand. (**C**) Heatmap showing the signal intensity of G4 structures and DRIP-seq in BJ + RAS cells, relative to the indicated genomic coordinates and the presence of QPSs marked by red arrows. In the case of the *KRAS* locus, these correspond to the well-characterized G4 regions referred to as ‘near’, ‘mid’, and ‘far’. (**D**) Metaplot of DRIP-seq and G4, RNA PolII Ser5 and BRCA1 ChIP-seq signals in BJ + RAS and BJ + RAS/HDAC4 cells within 6kb of “G-loop like” structures (R-loops co-occurring with G4 in non-template strand) identified in BJ + RAS cells. (**E**) The *in vitro* assay was performed by incubating equimolar amounts (800 pmol) of Cy5.5-labeled R-loops, G-loops containing the G4 motif “A” (ref. Fig. 3A) or DNA:RNA duplex (Cy5.5 labeling at 5′ of RNA) with increasing amounts of RNase H1. Native gel electrophoresis was performed to separate the various species of nucleic acids, which were schematized on the side. The fluorescence of the Cy5.5 labeled RNAs was acquired with the fluorescence reader and then the gel was stained with EtBr to detect the DNA duplexes with the transilluminator. (**F**) Histogram representing the ribonuclease efficiency of RNase H1 against synthetic R-loop and G-loop substrates (increasing amounts: 80, 160, and 400 pmol). Lanes corresponding to gel electrophoresis (in E) are indicated. Structures containing G4 in displaced DNA strand are in black (lanes 8–12). (**G**) Histogram representing the increase in fluorescence of Thioflavin T in the presence of substrates containing G4. Fluorescence emission was collected at 485 nm after excitation at 425 nm and expressed as increase of fluorescence quantum yield at 485 nm in the presence of G4-forming sequences in displaced DNA strand (lanes 8–12, increasing amounts: 80, 160, and 400 pmol). (**H**) Dot blot analysis using BG4 antibody to detect G4. Increasing amount of the indicated synthetic DNA:RNA structures (DNA:RNA heteroduplex not containing G4, R-loop not containing G4, G-loop with motif A, G-loop with 32R G4) were spotted on nitrocellulose film. RNA was Cy5.5 labeled at 5′. Cy5.5 fluorescence was used as loading control. (**I**) Fluorescence anisotropy binding assays showing the interaction between catalytically inactive RNase H1 (deadRNase H1) and the indicated nucleic acid substrates. Binding curves were fitted to determine the apparent dissociation constants (*K*_d_) for each substrate. In F and G, data are expressed as mean ± SD; *n* = 4. *P < 0.05, **P < 0.01, ***P < 0.05. Pairwise t-test was applied to indicated comparisons.

We first identified the most enriched motifs in G4-ChIP-seq from pre-RIS cells using Centrimo (MEME Suite) [13]. The top three enriched motifs were refined to optimal consensus sequences using G4-Hunter [75] (Fig. 3A). A synthetic DNA oligonucleotide corresponding to motif A (the one with the highest G4-forming potential according to G4-Hunter, indicated as “A” in the figures), folded in 100 mM KCl buffer, exhibited a melting temperature (Tm) of 64.9°C measured by absorbance at 295 nm (Supplementary Fig. S4A), consistent with stable G4 formation [76]. This was supported by thermal differential spectroscopy (TDS), which revealed negative band at 295 nm and positive bands at 275 nm, typical of quadruplex DNA (Supplementary Fig. S4B).

We then restricted our bioinformatic analysis to G4s enriched in BJ + RAS cells that matched these three optimal motifs and were located within 10 kb of an R-loop. Of these regions, 79.3% exhibited features compatible with G-loop formation, with the G4 located on the non-template strand either coinciding with or downstream of the TSS (Fig. 3B). The remaining 21.7% had G4s on the template strand (Fig. 3B), never co-occurring with R-loops but consistently located upstream of them, in agreement with previous reports on the incompatibility of G4s on template strand and R-loops [45, 71]. These two categories are exemplified in Fig. 3C, showing the endogenous loci of *KRAS* (marked by G4s in the promoter on the template strand and a R-loop downstream the TSS) and *PDXDC1* (configuration compatible with a G-loop). Given the higher prevalence of the latter configuration, we focused subsequent analyses on these “G-loop like” loci.

We examined the distribution of G4, R-loop (DRIP-seq), BRCA1 binding, and RNA Polymerase II CTD Ser5 phosphorylation signals within ± 3 kb of the putative G-loop regions in pre-RIS (BJ + RAS) and RIS-escaper (BJ + RAS/HDAC4) cells. We observed enrichment of G4 and DRIP-seq signals in pre-RIS cells, accompanied by reduced BRCA1 chromatin loading and unaltered RNA Pol II Ser5 levels (Fig. 3D). These findings suggest that accumulation or delayed resolution of “G-loop-like” structures correlates with defective BRCA1 recruitment, particularly in cells undergoing RIS.

To test whether G-loop-compatible configurations exhibit increased DNA:RNA hybrid stability and reduced RNase H1 accessibility, we synthesized and folded two types of R-loops: a canonical R-loop lacking G-rich regions and a “G-loop-like” R-loop containing a G4 identical to motif A. The assembly of R-loops and G-loops is described in detail in the Materials and Methods section. The RNA strand was 5'-labeled with Cy5.5 to monitor RNase H1 processing. Ethidium bromide (EtBr) staining was used to visualize nucleic acids predominantly in double-stranded conformation, due to its preferential intercalation between base pairs of duplex DNA and RNA [77]. Electrophoretic analysis revealed that G-loops were poorer substrates for RNase H1 compared with canonical R-loops (Fig. 3E). Ribonucleolytic cleavage fragments accumulated at 0.54 µM RNase H1 (1 unit) for canonical R-loops, whereas G-loops required a threefold higher RNase H1 concentration to initiate cleavage (Fig. 3E). Recent findings have demonstrated that, contrary to previous reports, RNase H1 exhibits not only endoribonucleolytic activity but also exoribonucleolytic activity [48]. The electrophoretic cleavage pattern observed is consistent with either endonucleolytic or exonucleolytic mechanisms. To strengthen this observation, we developed a fluorescence-based assay in which the RNA molecule is conjugated to a Cy5.5 fluorophore via a phosphodiester bond at 5′. A decrease in fluorescence intensity is therefore indicative of exonucleolytic degradation as well (Supplementary Fig. S4C). The data revealed significantly reduced RNase H1 activity on G-loops (Fig. 3F) and slower cleavage kinetics (Supplementary Fig. S4D). The folded G-loop structure contained a G-quadruplex, as demonstrated by using two orthogonal techniques. First, Thioflavin T staining, whose fluorescence increases upon binding to G4s [50], showed significantly stronger labeling of the synthetic G-loop compared with the G4-lacking R-loop (Fig. 3G); second, dot blot analysis (Fig. 3H) using the BG4 antibody confirmed the presence of G4 structures exclusively in the G-loop substrates [59]. Furthermore, the resistance of G-loops to RNase H1 cleavage is not sequence-dependent, as a similar reduction in cleavage kinetics was observed when the G4 element within the G-loop was replaced with a different sequence, namely the well-characterized 32R [78] G4 motif from KRAS (Supplementary Fig. S4E). The reduced susceptibility of G–loops to RNase H1–mediated cleavage, compared with canonical R–loops, appears to arise at least in part from diminished binding and/or substrate recognition by RNase H1. Fluorescence polarization assays performed with a catalytically inactive RNase H1 variant (dRNase H1, carrying the D210N mutation that abolishes metal–ion–dependent RNA hydrolysis without affecting binding affinity [61]) in the presence of increasing concentrations of DNA:RNA hybrids, R–loops, or G–loops revealed markedly higher affinity for DNA:RNA hybrids and R–loops than for G–loops, whereas ssRNA was bound only weakly (Fig. 3I). An orthogonal binding assay, in which dRNase H1 was incubated with increasing amounts of fluorescently labeled R–loops or G–loops followed by quantification of RNase H1–associated fluorescence, corroborated these findings: dRNase H1 consistently bound G–loops less efficiently than R–loops (Supplementary Fig. S4F).

Together, these results demonstrate that structures in which R–loops coexist with G–quadruplexes—here referred to as “G–loop–like” structures—exhibit intrinsic resistance to RNase H1 cleavage.

### Accumulation of R-loops in RIS correlates with a reduced ability of RNase H1 to form a complex with RPA

**Figure 4.**
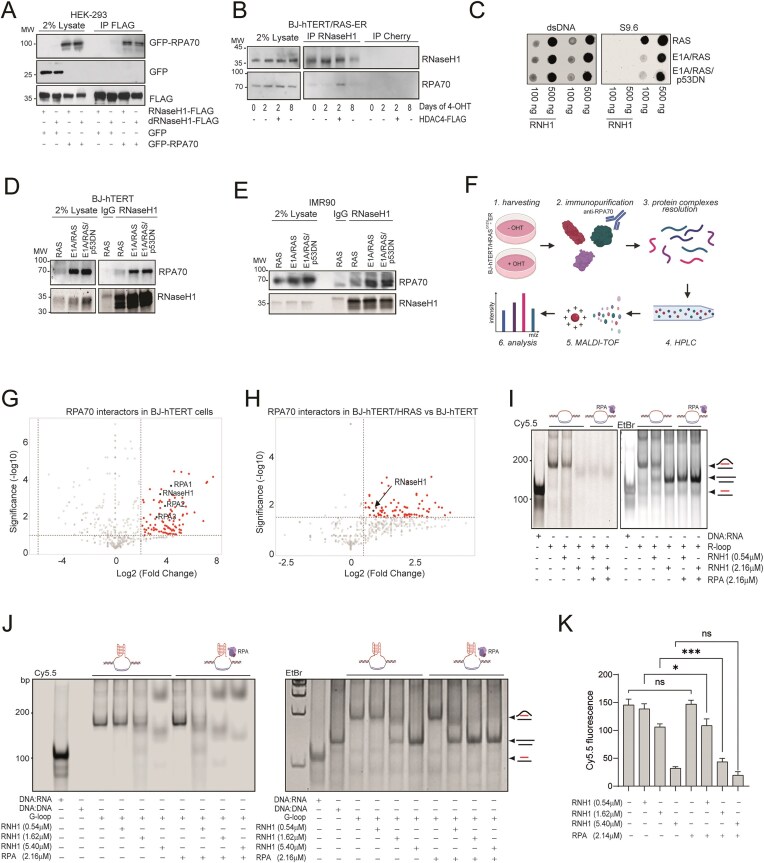
The accumulation of R-loops in OIS correlated with a reduced ability of RNase H1 to form a complex with RPA. (**A**) Around 293 cells were co-transfected with plasmid expressing GFP-RPA70 and RNase H1-FLAG as indicated. Immunoprecipitation was performed by using 1 μg of anti-FLAG antibody. 1/50 total lysate was included as input. (**B**) Co-IP experiment in BJ-hTERT/RAS-ER cells expressing or not HDAC4 as indicated, treated or not with 4-OHT to induce HRAS expression as indicated. Native lysates were immunoprecipitated with 1 μg anti-RNase H1 antibody or anti-Cherry antibody as a control. (**C**) Dot blot analysis using S9.6 antibody to detect R-loops and AE-2 antibody to detect dsDNA in BJ/hTERT cells stably expressing the indicated transgenes. Around 100 and 500 ng of nucleic acids extracted from the indicated cells were treated or not with 10u of RNase H and spotted on nitrocellulose film. (**D**) Co-IP experiment in BJ-hTERT cells expressing the indicated transgenes. Native lysates were immunoprecipitated with 1 μg anti-RNase H1 antibody or anti-IgG antibody as a control. 1/50 total lysate was included as input. (**E**) Co-IP experiment in IMR90 cells expressing the indicated transgenes. Native lysates were immunoprecipitated with 1 μg anti-RNase H1 antibody or anti-IgG antibody as a control. 1/50 total lysate was included as input. (**F**) Scheme of the experimental design for the quantitative identification of RPA70 interactors in BJ-hTERT/RAS-ER cells treated or not with 4-OHT for 2 days to reach or not a pre-senescent state. (**G**) Volcano plot showing the distribution of RPA70 interactors in BJ-hTERT/RAS-ER cells not induced to express HRAS^G12V^. In this graph, *x*-axis is plotted with log2 transformation of the fold difference between RPA70 and IgG IP; *y*-axis shows the-log10 transformation of the *P*-values for the identified proteins. RPA70, RPA32, RPA14, RNase H1 are highlighted. (**H**) Volcano plot showing the distribution of RPA70 interactors in BJ-hTERT/RAS-ER cells induced to express HRAS^G12V^ with respect to the same cells not induced. In this graph, *x*-axis is plotted with log2 transformation of the fold difference between BJ + RAS and BJ-RAS; *y*-axis shows the -log10 transformation of the *P*-values for the identified proteins. (**I, J**) The *in vitro* assay was performed by incubating Cy5.5-labeled R-loops (I), G-loops (J) or DNA:RNA duplex (labeled both on RNA at 5′) with the indicated amounts of RPA complex or RNase H1. Native gel electrophoresis was performed to separate the various species of nucleic acids, which were schematized on the side. The fluorescence of the Cy5.5 labeled RNAs was acquired with the fluorescence reader and then the gel was stained with EtBr to detect the DNA duplexes with the transilluminator. (**K**) Histogram representing the ribonuclease efficiency of RNase H1 against G-loop substrates in the presence or absence or RPA. Data are expressed as mean ± SD; *n* = 4. *P < 0.05, **P < 0.01, ***P < 0.05. Pairwise t-test was applied to indicated comparisons.

The heterotrimeric RPA complex (RPA1/RPA70, RPA2/RPA32, RPA3/RPA14) facilitates the assembly of the ATR-ATRIP complex on ssDNA [79]. At the same time, through its binding to non-template DNA strand, the RPA complex instructs RNase H1 to recognize R-loops [49], it reinforces RNase H1’s endoribonuclease activity and stimulates its exoribonuclease activity [48]. This interaction was reported to be direct and to involve RPA70 [49]. We hypothesized that the accumulation of R-loops at γH2AX +sites in cells undergoing RIS could be due to their slowed processing by RNase H1 as a result of inefficient instruction by RPA70. First, we detected positive binding between RPA70 and RNase H1 and dRNase H1 in 293 cells co-expressing both targets (Fig. 4A), confirming a previous report [49]. Next, we confirmed complex formation at endogenous levels (Fig. 4B). IP of RNase H1 led to co-IP of RPA70 in cells escaping OIS (BJ + RAS/HDAC4), while only weak binding was detected in pre-OIS state (BJ + RAS, Fig. 4B). Comparable results were obtained by immunoprecipitating RPA70 in reverse immunoprecipitation experiments (Supplementary Fig. S5A).

To strengthen these findings, we employed an alternative condition of OIS escape, based on the co-expression of RAS with E1A and a dominant-negative form of p53 (*TP53^175H^*) [13, 65]. Under this condition, senescence escape correlated with a decrease in DNA:RNA hybrids detected by dot blot using the S9.6 antibody (Fig. 4C). We confirmed the superior binding between RPA70 and RNase H1 both in BJ/E1A/RAS and BJ/E1A/RAS/P53DN (*TP53^175H^*) with respect to BJ/RAS (Fig. 4D) and in IMR90/E1A/RAS and IMR90/E1A/RAS/P53DN with respect to IMR90/RAS (Fig. 4E). The increased binding between RPA70 and RNase H1 correlated with lower levels of R-loops detected in dot blot (Fig. 4C).

To further confirm these data, we identified RPA70 interactors by mass-spectrometry in BJ-hTERT cells expressing *HRAS^G12V^* for two days (pre-OIS state) or in normally proliferating BJ-hTERT cells not expressing *HRAS^G12V^* (Fig. 4F). We identified 167 interactors of RPA70 in BJ-hTERT proliferating cells [log2(fold-change) > 2, *P* < 0.1], including RPA32 and RPA14, demonstrating that the heterotrimeric complex is formed in these cells (Fig. 4G). Importantly, we identified by mass-spectrometry well-known interactors of RPA (Supplementary Table S5), including RNase H1 (Fig. 4G). Around 48 out of these 167 interactors, among which RNase H1, were found to be complexed with RPA70 in BJ-hTERT proliferating cells but not, or significantly less, in BJ-hTERT/HRAS^G12V^ (Fig. 4H). These 48 hits [log2(fold-change) > 0.5, p < 0.1] (Supplementary Table S6) are involved in RNA processing and in regulating DNA damage response (Supplementary Fig. S5B). This indicates a partial decomplexation of RPA70 from its partners, including RNase H1, in pre-senescent cells. Importantly, the assembly of the trimeric RPA complex in pre-senescent cells was not altered compared with normal proliferating ones (Supplementary Table S5).

To test whether the RPA complex enhances RNase H1-mediated R-loop processing, we performed an *in vitro* assay in which we incubated the synthetic R-loop described before, containing the RNA strand complementary to DNA fluorescently labeled with Cy5.5, with increasing amounts of RNase H1 in the presence or absence of the purified recombinant RPA complex (Fig. 4I). Native gel electrophoresis of the reaction products showed enhanced cleavage of the synthetic R-loop by RNase H1 in the presence of the RPA complex, with complete processing already achieved with 540nM RNase H1, whereas the same amount of enzyme alone (in the absence of RPA) resulted in approximately 50% processing, as shown by residual Cy5.5 fluorescence (Fig. 4I left) and the signal after UV exposure of the same gel stained with the double-stranded intercalator ethidium bromide (Fig. 4I right).

RPA was reported to bind with similar affinities to both ssDNA and G4s (with a kD of ∼1 nM, whereas the kD for dsDNA is in the micromolar range) [80], and to exhibit G4 unwinding activity [81, 82] that depends on G4 topology and loop architecture [80, 83]. We therefore tested whether the RPA complex could stimulate the RNase H1 activity also on G-loops. To this end, we repeated the previously described *in vitro* assay using a synthetic G-loop containing the G4 motif named ‘A’. In this context as well, the RPA complex markedly enhanced RNase H1 activity (Fig. 4J). In the presence of RPA, RNase H1 activity was increased 3-folds, as processing observed with 1.62 µM RNase H1 was equivalent to that obtained with 5.40 µM RNase H1 alone, in the absence of RPA (Fig. 4K). The reduction in Thioflavin T staining indicated partial unwinding of G4s, which was more consistent for DNA G4s and less for the G-loop (Supplementary Fig. S5C). We cannot exclude that this unwinding, although partial, contributes to the resolution of G-loops stimulated by RPA.

Overall, these data showed that:

- the RPA complex promotes R-loop processing by RNase H1;
- the RPA complex promotes G-loop processing by RNase H1;
- the binding between RPA and RNase H1 is weaker in cells entering RIS than in normally proliferating cells and in cells escaping RIS; this could explain the abnormal accumulation of R-loops and G-loops-like structures observed in cells undergoing OIS.

### Hyperphosphorylation of RPA32 leads to disassembly of the RPA/RNase H1 complex

**Figure 5.**
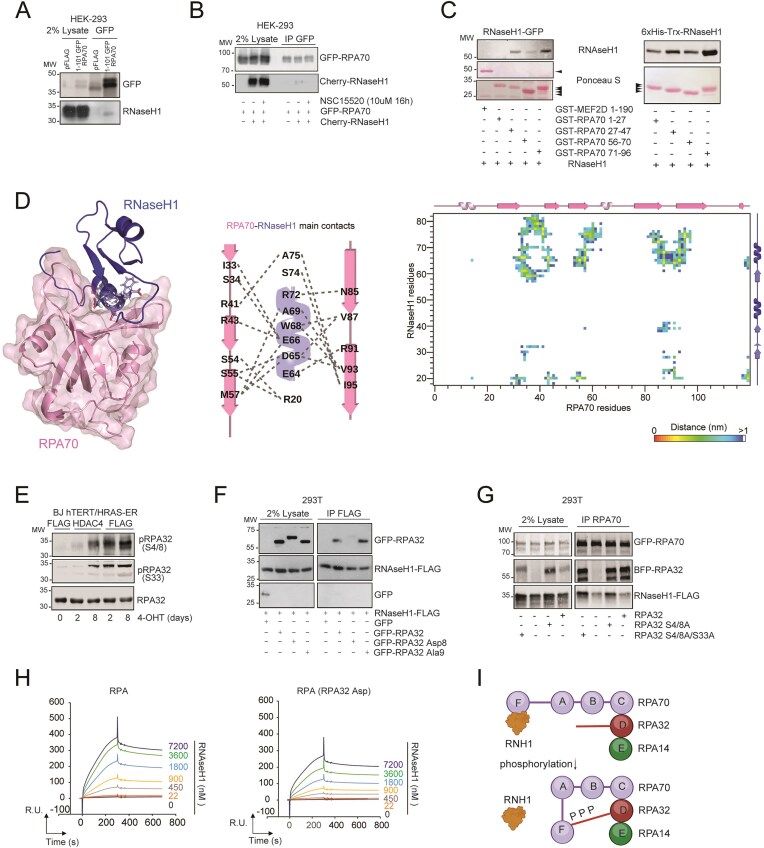
Hyperphosphorylation of RPA32 leads to disassembly of the RPA/RNase H1 complex. (**A**) 293 cells were transfected with a plasmid expressing a deletion of GFP-RPA70 or a control empty plasmid, as indicated. Immunoprecipitation was performed by using 1 μg of anti-GFP antibody. 1/50 total lysate was included as input. (**B**) Around 293 cells were co-transfected with a plasmid expressing GFP-RPA70 and Cherry-RNase H1, as indicated. At 36 h from transfection, cells were treated or not with NSC15520 for 16 h, then harvested and subjected to immunoprecipitation by using 1 μg of anti-GFP antibody. 1/50 total lysate was included as input. (**C**) GST pull-down was performed by incubating GST-tagged fragments of recombinant RPA70, or MEF2D as a negative control, with RNase H1-GFP (left, obtained from transfected 293 cells) or His-tagged recombinant human RNase H1 (right, obtained from bacteria), as indicated. (**D**) *In silico* structural prediction of the RPA70-RNase H1 complex. Left: graphical representation of the binding conformation between the OB domain of RPA70 (pink) and the RNA:DNA hybrid-binding domain of RNase H1 (blue) corresponding to the most representative configuration (the center of the biggest cluster) in the molecular dynamics trajectory. Center: diagram of the principal interactions in the RPA70-RNase H1 complex. Right: map of the average minimum distances between residues of RPA70 and RNase H1. (**E**) Immunoblot analysis of the indicated proteins in BJ-hTERT/RAS-ER cells, expressing the indicated transgenes and treated or not as indicated with 4-OHT (1µM). (**F**) 293 cells were co-transfected with plasmids expressing RNase H1-FLAG and the wild type, phosphodead (Ala9) or phosphomimetic (Asp8) mutants of RPA32, as indicated. Immunoprecipitation was performed by using 1 μg of anti-FLAG antibody. 1/50 total lysate was included as input. (**G**) Around 293 cells were co-transfected with plasmids expressing RNase H1-FLAG, BFP-RPA32 (wild type, phosphodead (Ala 9) or phosphomimetic (Asp 8)) and GFP-RPA70, as indicated. Immunoprecipitation was performed by using 1 μg of anti-RPA70 antibody. 1/50 total lysate was included as input. (**H**) SPR experiment performed by immobilizing RPA complexes (containing wild-type RPA32, left, or the phospho-mimicking mutant of RPA32, right) on a Biacore CM5 chip surface via amine-coupling. Multiple concentrations of RNase H1 (0–7200 nM) were injected on CM5 SPR. Sensorgrams confirmed the decrease in interaction between the RPA complex and RNase H1 with RPA Asp. (**I**) Scheme illustrating the bending of the F-domain of RPA70 on RPA32 when the latter is phosphorylated thus displacing RNase H1.

RPA heterotrimeric complex contains multiple DNA-binding domains involved in ssDNA binding [84]. The *N*-terminal OB domain of RPA70 is not involved in ssDNA-binding activity but allows the interaction with multiple DNA damage signaling factors [85]. We demonstrated the interaction between the *N*-terminal region of RPA70 [1–86, 55, 87–100] and RNase H1 by co-immunoprecipitation in 293 cells co-expressing both the targets (Fig. 5A). This result corroborated the involvement of RPA70 in the interaction of RPA with RNase H1 [49]. As a further confirmation, 15-carboxy-13-isopropylatis-13-ene-17,18-dioic acid (NSC15520), a small molecule described to bind RPA70 *N*-terminus and interfere with the binding to partner proteins [86], was effective in displacing GFP-RPA70 and Cherry-RNase H1 in HEK-293 cells co-expressing both the proteins (Fig. 5B). To better define the minimal domains of RPA70 required for the interaction with RNase H1, deletion mutants of GST-RPA70 were expressed in bacteria, purified and used in GST-pull down-experiments (Fig. 5C). Two anti-parallel β-sheets and their connecting β-turns (amino acids 27–47 and 71–96) were identified as being directly involved in the interaction (Fig. 5C). The direct involvement of β-sheets and β-turns in the interaction with partners of the DNA damage response is a common feature of RPA70 [55].

*In silico* prediction of the binding conformation between the OB domain of RPA70 and the RNA:DNA hybrid-binding domain of RNase H1 supported the interaction model proposed in Fig. 5D. Indeed, the extensive exploration of a set of RPA70-RNase H1 possible complexes generated by docking led to the identification of three promising candidates (Supplementary Fig. S6A). The subsequent screening by means of molecular dynamics (MD) simulations allowed us to pinpoint the most stable binding conformation (Supplementary Fig. S6B). In this conformation, the acidic α-helix of RNase H1 hybrid-binding domain primarily interacts with the residues located in the RPA70 groove formed by the two anti-parallel β-sheets and their connecting β-turns (Fig. 5D). The computational results support a model in which RNase H1 residues from the neighboring disordered regions are essential for the stabilization of binding (Supplementary Fig. S6B and Fig. 5D).

The availability of the OB domain of RPA70 for interaction with its DNA damage response partners is tightly regulated at the post-translational level [55, 87]. One of the strongest regulations is the hyperphosphorylation of RPA2/RPA32, which forces interaction with the *N*-terminal domain of RPA70, thereby altering the canonical folding of the heterotrimeric complex and the interaction with important molecular partners, including those involved in end resection [87]. Consistently, RPA32 hyperphosphorylation characterized HRAS-expressing cells that enter RIS compared with those co-expressing *HRAS* and *HDAC4* that escaped OIS, in particular at day 2 of HRAS induction (Fig. 5E).

We therefore tested whether a phosphodead mutant (Ala9: S8, S11, S12, S13, S23, S29, S33, S39A, and T21A) or a phosphomimetic mutant (Asp8: S8, S11, S12, S13, S23, S29, S33D, and T21D) of RPA32 would impact the binding of the RPA complex to RNase H1. As hypothesized, the expression of phosphomimetic mutant of RPA32 in HEK-293 cells lead to RNase H1 detachment from the complex (Fig. 5F and Supplementary Fig. S7A). Moreover, in cells co-expressing RPA70, RPA32 and RNase H1, the two phosphodead mutants of RPA32 (S4/8A and S4/8/33A) were equally powerful in boosting the interaction of RPA70 with RNase H1 (Fig. 5G). To further confirm this finding, we employed Surface Plasmon Resonance (SPR). For this purpose, two RPA complexes, containing wt (defined as “RPA”) or phosphomimetic RPA32 (Asp 8, defined as “RPA Asp”), were purified (Supplementary Fig. S7B) and captured on a Biacore CM5 chip surface via amine-coupling (Supplementary Fig. S7C). Multiple concentrations of RNase H1 (0-7200 nM) were injected on CM5 SPR, as reported in sensorgrams depicted in Fig. 5H. The interaction rate constants of the RPA and RPA Asp with RNase H1 were calculated from three independent cycles of measurements and by fitting the sensorgrams to 1:1 Langmuir Binding model. From each binding-release profile, the association (ka) and dissociation (kd) rate constants were determined. The equilibrium dissociation constant *K*_D_ (*K*_D _= *k*_d_/*k*_a_) for RPA was 1.54 ± 0.734 × 10^−7^M and for RPA Asp was 2.42 ± 1.06 × 10^−7^M. Overall, the SPR experiments confirm: i) the binding between the RPA complex and RNase H1 using an orthogonal method compared to co-immunoprecipitation and GST pull-down; ii) a higher affinity of the non-phosphorylated form of the RPA complex for RNase H1 also *in vitro* (Fig. 5I). These data suggest a model in which, in the presence of phosphorylated RPA32, the OB *N*-terminal domain of RPA70 folds onto RPA32, weakening the interactions of RPA70 *N*-term with its partners, including RNase H1, in analogy to what has been proposed for explain the dissociation of the hyperphosphorylated RPA complex from EXO1, BLM, or DNA2 (Fig. 5I) [87].

### The disassembly of the RPA/RNase H1 complex due to hyperphosphorylation of RPA32 slows down the processing kinetics of R-loops and G-loops

**Figure 6.**
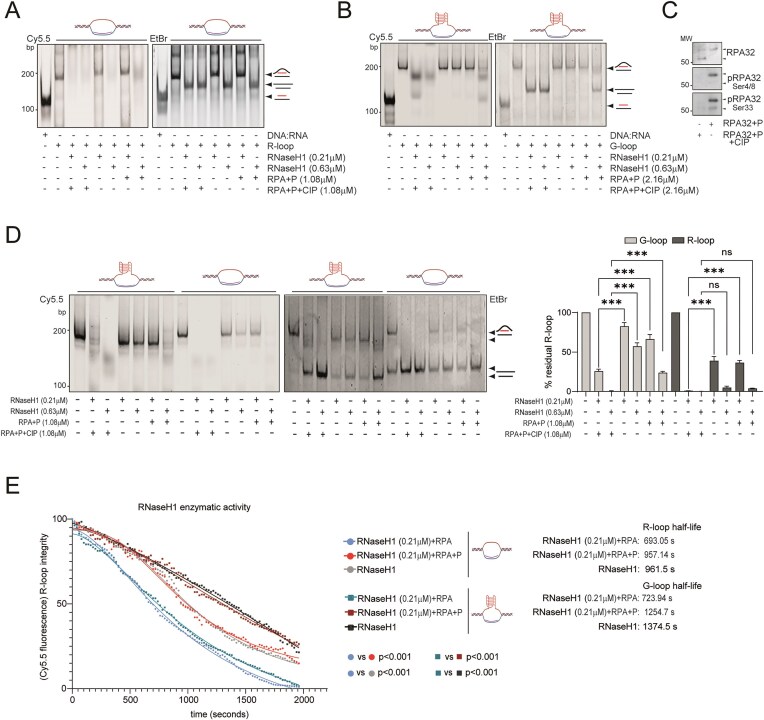
Hyperphosphorylation of RPA32 leads to disassembly of the RPA/RNase H1 complex and slows down the processing kinetics of R-loops and G-loops. (**A, B**) The *in vitro* assay was performed by incubating for 20′ Cy5.5-labeled R-loops (A), G-loops (B) or DNA:RNA duplex (all labeled on RNA at 5′) with the indicated amounts of RPA complex or RNase H1. Native gel electrophoresis was performed to separate the various species of nucleic acids, which were schematized on the side. The fluorescence of the Cy5.5 labeled RNAs was acquired with the fluorescence reader and then the gel was stained with EtBr to detect the DNA duplexes with the transilluminator. RPA complex was phosphorylated *in vitro* and then dephosphorylated or not with calf intestine phosphatase (CIP), as indicated. (**C**) Immunoblot on recombinant RPA32 subjected to *in vitro* phosphorylation and dephosphorylation. The indicated phospho-specific antibodies were used. (**D**) The *in vitro* assay was performed by incubating Cy5.5-labeled R-loops, G-loops or DNA:RNA duplex (all labeled on RNA at 5′), as illustrated, with the indicated amounts of RPA complex (containing or not phosphorylated and dephosphorylated RPA32) or RNase H1, as indicated. Data are expressed as mean ± SD; *n* = 3. (**E**) Plot of the ribonuclease kinetics of RNase H1 against synthetic R-loops or G-loops as schematized, in the presence or absence of the RPA complex containing either phosphorylated or dephosphorylated RPA32. In D and E, statistics are provided as pairwise comparisons. *P < 0.05, **P < 0.01, ***P < 0.05.

To determine whether phosphorylation of RPA32 reduces the catalytic activity of RNase H1, we performed the previously described *in vitro* assay using a Cy5.5-labeled synthetic R-loop substrate. Increasing amounts of RNase H1 were incubated with the substrate in the presence or absence of the purified recombinant RPA complex in which RPA32 was phosphorylated *in vitro* (RPA32 + P) or dephosphorylated by calf intestinal phosphatase (RPA32 + P + CIP) (Fig. 6A, C). Native gel electrophoresis of the reaction products showed enhanced cleavage of the synthetic R-loop by RNase H1 in the presence of the dephosphorylated RPA complex (Fig. 6A). In contrast, the same amount of RNase H1 alone was ineffective or minimally effective in the presence of phosphorylated RPA complex, as shown by both the quantification of residual Cy5.5 fluorescence and the signal after UV exposure of the same gel stained with EtBr (Fig. 6A). Similarly, RPA complex containing the dephosphorylated form of RPA32 promoted the processing of a G-loop by RNase H1, whereas the RPA complex containing the hyperphosphorylated form of RPA32 significantly decreased the stimulatory effect of RPA on RNase H1 ribonuclease activity (Fig. 6B). Fig. 6C shows the phosphorylation status of RPA32 and its dephosphorylation following treatment with CIP phosphatase of the RPA complexes used in Fig. 6A, B.

We then compared the ribonuclease activity of RNase H1 on equimolar amounts of synthetic R-loop and G-loop substrates in the presence of RPA complexes containing either phosphorylated or dephosphorylated RPA32, or in the absence of RPA. As shown in Fig. 6D, RNase H1 was less efficient on G-loops than on R-loops in the absence of RPA or in the presence of RPA containing phosphorylated RPA32. However, addition of RPA containing dephosphorylated RPA32 abolished this difference, rendering RNase H1 equally efficient on G-loops (Fig. 6D).

This observation was further confirmed by a kinetic analysis of RNase H1 ribonuclease activity on both substrates in the presence or absence of RPA complexes containing either phosphorylated or dephosphorylated RPA32 (Fig. 6E). Consistently, the cleavage kinetics of RNase H1 on R-loops and G-loops were comparable in the presence of RPA containing dephosphorylated RPA32 (Fig. 6E).

Overall, these data confirm a slower cleavage kinetics of G-loops compared with R-loops by RNase H1 and that a functional RPA complex abolishes this difference by promoting RNase H1 activity both on R-loops and G-loops substrates.

### RPA70 overexpression facilitates the recognition of R-loops by RNase H1 and this correlates to partial OIS escape

**Figure 7.**
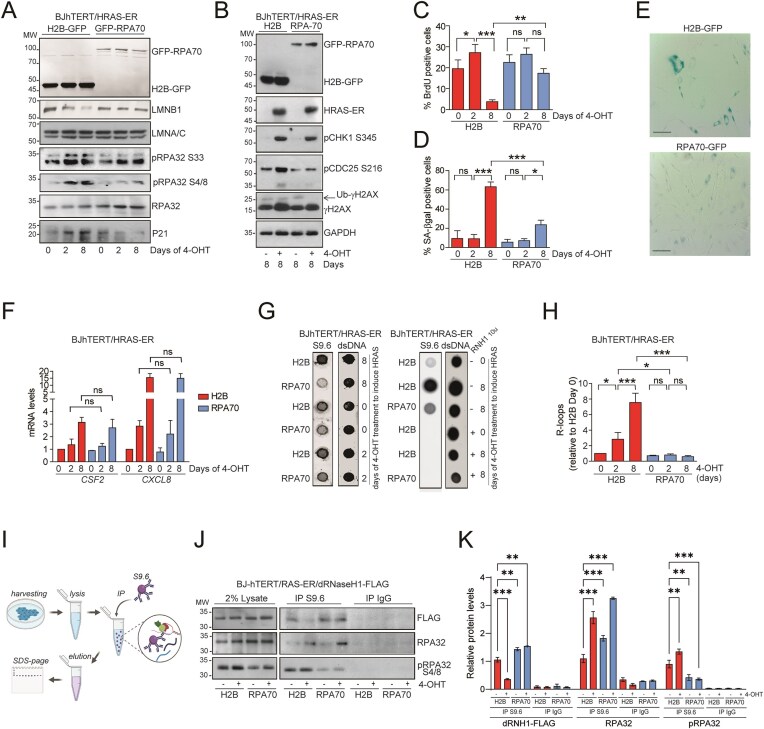
RPA70 overexpression in RAS-expressing cells leads to reduced R-loop levels and partial escape from oncogene-induced senescence. (**A, B**) Immunoblot analysis of the indicated proteins in BJ-hTERT/RAS-ER cells, expressing H2B-GFP or GFP-RPA70 as indicated and treated or not with 4-OHT (1 µM). (**C, D**) Quantification of SA-β-gal-positive cells and S-phase entering cells (BrdU assay, BrdU pulse of 3 h) in BJ-hTERT/RAS-ER cells, expressing H2B-GFP or GFP-RPA70 as indicated and treated or not with 4-OHT (1 µM). (**E**) Representative microscopic images of SA-β-gal-stained BJ-hTERT/RAS-ER H2B-GFP or GFP-RPA70 cells at 8 days after 4-OHT treatment (bar = 50 µm). (**F**) mRNA expression levels of the indicated SASP genes in BJ-hTERT/RAS-ER H2B-GFP or GFP-RPA70 cells at 0, 2, and 8 days after 4-OHT treatment. Mean ± SD; *n* = 3. Pairwise t-test was applied to indicated comparisons. (**G**) Dot blot analysis using S9.6 antibody to detect R-loops and AE-2 antibody to detect dsDNA. Around 250 ng of nucleic acids extracted from BJ-hTERT/RAS-ER H2B-GFP or GFP-RPA70 cells grown for 0, 2, and 8 days in the presence or absence of 4-OHT were treated or not with 10 U of RNase H and spotted on nitrocellulose membrane. (**H**) Quantification of R-loop signals obtained in dot blot described in G. Mean ± SD; *n* = 3. Dunn’s Multiple Comparison Test was applied to indicated comparisons. (**I**) Schematic illustration of the procedure for purifying proteins associated with R-loops in lysates obtained from BJ-hTERT/RAS-ER H2B-GFP or GFP-RPA70 cells expressing dRNase H1, treated or not for 2 days with 4-OHT. (**J**) Immunoblot analysis of the indicated proteins co-purified with R-loops from indicated cells treated or not for 2 days with 4-OHT, as indicated. (**K**) Quantification of the indicated proteins co-purified with R-loops from indicated cells treated or not for 2 days with 4-OHT, as indicated. In C, D, F, H, and K, data are expressed as mean ± SD; *n* = 3. Dunn’s Multiple Comparison Test was applied to indicated comparisons. *P < 0.05, **P < 0.01, ***P < 0.05.

Cells exposed to high levels of replication stress deplete the RPA pools in the nucleus and accumulate unrepaired DSBs at later time points [88]. Overexpression of RPA complex (stoichiometric levels of RPA1/RPA70, RPA2/RPA32 and RPA3/RPA14) reduced the formation of unprotected ssDNA and slowed the accumulation of replication stress following hydroxyurea treatment or ATR inhibition [88]. However, as previously reported [89], overexpression of RPA70 has minimal effects on overall RPA complex activity. We therefore hypothesized that RPA70 overexpression could be used to assess whether it promotes RNase H1 engagement and, consequently, enhances its ribonuclease activity toward R-loops and G-loops. To prove this, we generated BJ-hTERT/HRAS-ER cells overexpressing GFP-RPA70 and H2B-GFP, as a control. It has been reported that GFP-RPA70 fusion does not affect RPA molecular functions [90] and that H2B-GFP does not induce heterochromatinization and does not affect OIS entrance/escape [13]. Following *HRAS^G12V^* induction, RPA70 overexpressing cells were characterized by decreased phosphorylation levels of RPA32 (S33 and S4/8), γH2AX, ubiquitylated γH2AX and phosphorylated CDC25 (S216) compared with control cells, but similar levels of active CHK1 (S345) (Fig. 7A, B). RPA70 overexpressing cells were also resistant to LMNB1 degradation after *HRAS^G12V^* induction (Fig. 7A), accumulated decreased levels of CDKN1A/P21 (Fig. 7A), kept on proliferating (Fig. 7C) and a significant proportion of them escaped senescence entry (Fig. 7D, E). Notably, no difference was observed in terms of expression of two SASP markers, *CSF2* and *CXCL8* (Fig. 7F), suggesting that overexpression of RPA70 did not alter the SASP program controlled by *HRAS* [7]. Remarkably, RPA70 overexpressing cells accumulated lower amounts of DNA:RNA hybrids compared with control cells (Fig. 7G, H). To prove that RPA70 overexpression increased the recruitment of RNase H1 to R-loops, we expressed dRNase H1 in BJ-hTERT/HRAS/H2B-GFP and GFP-RPA70 cells, that were or were not induced to express *HRAS* for 8 days. Then we pulled down the R-loops by using the S9.6 antibody and evidenced by SDS-page and immunoblotting the relative quantities of the RPA complex and RNase H1 associated with the R-loops (Fig. 7I). Despite comparable levels of dRNase H1 in the input, we observed higher levels of dRNase H1 and lower levels of phosphorylated RPA32 in the pull-down performed in cells overexpressing HRAS and GFP-RPA70 compared to HRAS/H2B-GFP cells (Fig. 7J, K). Overall, these data suggest that overexpression of RPA70 may be functional in re-educating RNase H1 to recognize and process R-loops.

### Genome-wide analysis reveals loss of RNase H1 activity at RPA32-hyperphosphorylated R-loop–enriched regions in pre-RIS cells

**Figure 8.**
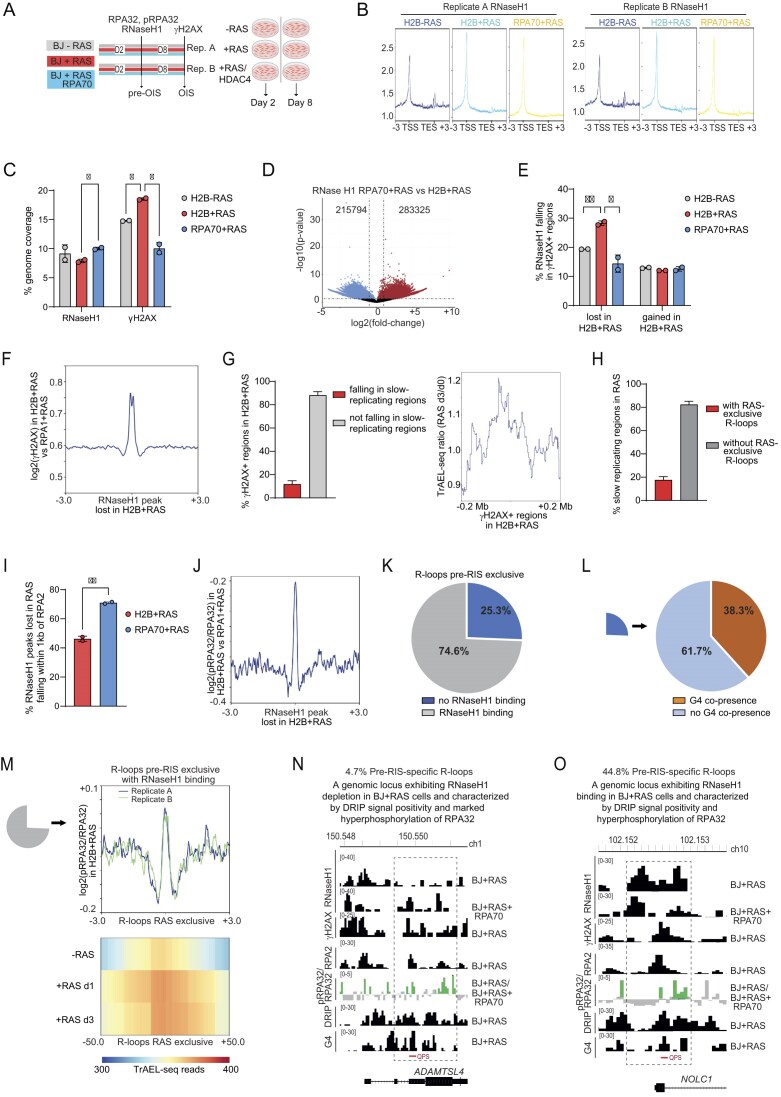
Genome-wide analysis reveals loss of RNase H1 activity at RPA32-hyperphosphorylated R-loop–enriched regions in pre-RIS cells. (**A**) Schematic representation of the ChIP-seq and DRIP-seq experiments performed in the indicated cells at the indicated time after 4-OHT treatment. (**B**) Metaplots of RNase H1 ChIP-seq levels in the indicated cells, with respect to TSS and TES. (**C**) Histogram representing the percentage of genome coverage of γH2AX and RNase H1 enriched peaks in the indicated cells. (**D**) Volcano plot showing RNase H1 peaks that are significantly enriched or depleted in the comparison between the RPA70 + RAS and the H2B + RAS cells. (**E**) Histogram showing the percentage of γH2AX + genomic regions overlapping with areas of RNase H1 binding loss in H2B + RAS cells compared with the RPA70 + RAS condition, and vice versa. (**F**) Metaplot showing the log2 ratio of γH2AX signal in H2B + RAS with respect to RPA70 + RAS cells within 6kb around the RNase H1 peaks lost in H2B + RAS cells. (**G**) Histogram and metaplot showing the percentage of γH2AX + genomic regions in H2B + RAS cells overlapping with genomic regions exhibiting increased TrAEL-seq read density in IMR90 RAS (≥1.5-fold enrichment after 3 days of RAS expression relative to day 0), consistent with replication fork pausing or altered fork dynamics (GSE299123). (**H**) Histogram showing the percentage of TrAEL-seq–enriched regions in HRAS-expressing cells overlapping with genomic loci characterized by R-loop accumulation in BJ + RAS pre-RIS cells. (**I**) Histogram showing that RNase H1 peaks lost in H2B + RAS cells are associated with a depletion of RPA32 binding compared to RPA70 + RAS cells. (**J**) Metaplot of log2 ratio of RPA32 phosphorylation rate (pRPA32/RPA32) signal in H2B + RAS with respect to RPA70 + RAS cells within 6kb around the RNase H1 peaks lost in H2B + RAS cells. (**K**) Venn diagram showing that 25.3% of R-loops exclusive to the pre-RIS condition do not exhibit overlap (1 nucleotide) of RNase H1 binding in H2B + RAS cells, while 74.6% are characterized by RNase H1 association. (**L**) Venn diagram showing that 25.3% of R-loops exclusive to the pre-RIS condition that do not exhibit overlap (≥1 nucleotide) with RNase H1 binding in H2B + RAS cells show overlap with 38.3% of G4 regions. (**M**) Metaplot showing the hyperphosphorylation of RPA32 in H2B + RAS cells with respect to RPA70 + RAS within 6 kb around the center of the peaks of R-loops exclusive to the RIS condition and characterized by RNase H1 binding. For reference, a heatmap depicting the TrAEL-seq signal in IMR90 cells expressing RAS at the indicated time points is shown. (**N, O**) Distribution of R-loops, G4s, γH2AX and pRPA32 phosphorylation rate in RIS (BJ + RAS) and RIS-escape achieved by RPA70 overexpression (RPA70 + RAS). QPS are indicated. In C, E, G, H, I data are expressed as mean ± SD; two independent sequencing each coming from three biological replicates. *P < 0.05, **P < 0.01. Pairwise t-test was applied to indicated comparisons.

To investigate the effects of forced RNase H1 recruitment achieved through RPA70 overexpression, we analyzed three cell models: H2B − RAS (normally proliferating), H2B + RAS (pre-RIS state), and RPA70 + RAS (RIS-escaper cells). In these models, we performed genome-wide mapping of γH2AX and RNase H1, as well as of genomic loci exhibiting RPA32 hyperphosphorylation, quantified as the ratio of phosphorylated RPA32 (Ser4/8) to total RPA32 (Fig. 8A). RNase H1 showed a peak distribution at TSS and much less pronounced at TES (Fig. 8B). Phosphorylation of RPA32 and γH2AX was observed to decrease in the promoter region and increase along the gene bodies, showing similar profiles across the three cell models, although with varying intensities (Supplementary Fig. S8A, S8B). Importantly, in the RIS condition (H2B + RAS), γH2AX signal levels were higher than in the other conditions, while the genome covered by enriched RNase H1 peaks were higher in cells escaping RIS (RPA70 + RAS) (Fig. 8C).

We next identified RNase H1 peaks exhibiting preferential binding in RIS-escaper cells (RPA70 + RAS) compared with the pre–RIS state (H2B + RAS). Differential binding analysis was performed using DiffBind v3.18.0 [91], applying a cutoff of |log₂(fold change)| > 1 and an adjusted p–value < 0.05. Principal component analysis (PCA) showed a clear separation between the two sample groups (Supplementary Fig. S8C). This analysis revealed 283 325 RNase H1 peaks displaying exclusive or enriched binding in RPA70 + RAS relative to H2B + RAS (hereafter “RNase H1 peaks lost in H2B + RAS”) and 215 794 peaks preferentially associated with the pre-RIS condition (hereafter “RNase H1 peaks gained in H2B + RAS”) (Fig. 8D and Supplementary Fig. S8D). Importantly, RNase H1 peaks lost in the pre–RIS condition were predominantly enriched within γH2AX–positive regions (Fig. 8E). This pattern suggests that γH2AX accumulation in RIS cells may arise at genomic loci where RNase H1 binding is diminished. Consistent with this idea, expressing the γH2AX signal as the log₂ ratio between RIS and RIS–escape conditions across these specific regions further confirmed an increase in γH2AX signal when RNase H1 binding was lost (Fig. 8F). These data suggest that γH2AX accumulation in H2B + RAS cells in respect to RPA70 + RAS cells occurs at sites where RNase H1 binding is lost and R-loops accumulate. Since R-loops are known to promote transcription–replication conflicts [92], we investigated whether R-loops accumulation correlates with replication fork pausing or slowing-down in pre-RIS cells. Recently, increased TrAEL-seq [93] read density has been reported to mark genomic regions associated with replication fork pausing or altered fork dynamics [94]. To investigate this, we reanalyzed TrAEL-seq data obtained from IMR90 fibroblasts expressing *HRAS^G12V^*-ER (GSE299123) and identified genomic regions exhibiting increased TrAEL-seq read density in pre-senescent cells expressing RAS for 3 days (defined by a ≥1.5-fold enrichment in TrAEL-seq read density compared with normally proliferating control cells at day 0 of RAS expression), consistent with fork pausing or altered replication dynamics. Slightly more than one tenth (10.8%) of γH2AX-positive regions in H2B + RAS cells overlapped with regions exhibiting increased TrAEL-seq signal (Fig. 8G). Conversely, 18.9% of these TrAEL-seq–enriched regions overlapped with genomic loci selectively accumulating R-loops in RAS-expressing cells (Fig. 8H). While this analysis cannot distinguish between fork slowing, transient pausing, or fork collapse, it supports the interpretation that R-loop accumulation in pre-RIS cells is associated with altered replication fork dynamics, consistent with observations in other contexts [34, 92].

Strikingly, we found that 58% of RNase H1 peaks lost in H2B + RAS cells (pre-RIS condition) were located in regions depleted of RPA32 binding, while RPA32 and RNase H1 co-localization within 1kb was generally preserved in RPA70 + RAS cells (Fig. 8I). More notably, in H2B + RAS cells compared with RPA70 + RAS cells, we observed an increased signal of phosphorylated RPA32 at the sites where RNase H1 binding was lost (Fig. 8J). These findings corroborate our previous molecular biology and biochemical data at the genomic level, confirming the loss of co-association between phosphorylated RPA32 peaks and RNase H1.

To further substantiate this concept, we intersected the peaks corresponding to R-loops identified as exclusive to the pre-RIS condition (Fig. 2A) with RNase H1 binding sites in the H2B + RAS and RPA70 + RAS conditions. This analysis revealed that 25% of the R-loops that accumulated exclusively in the pre-RIS condition following HRAS expression were associated with a loss of RNase H1 binding in H2B + RAS cells in respect to RPA70 + RAS cells (Fig. 8K). Thus, for at most one quarter of these R-loops, their accumulation could be attributed to impaired recognition by RNase H1. Notably, 38.3% of these R-loops co-mapped with G4-forming sequences, suggesting that they represented G-loop–like structures (Fig. 8L). Importantly, in the remaining 75% of genomic regions characterized by R-loop accumulation in the pre-RIS condition and concomitant RNase H1 binding, we observed hyperphosphorylation of RPA32 in H2B + RAS compared with RPA70 + RAS cells in correspondence with increased TrAEL-seq reads, compatible with altered replication fork dynamics in these genomic regions under RAS expression (Fig. 8M).

Although indirect, these results further supported the evidence for a reduction in RNase H1 ribonuclease activity in the presence of an RPA complex containing phosphorylated RPA32 (Fig. 8M), in agreement with our *in vitro* observations (Fig. 6A–D).

In Fig. 8N, O illustrate two distinct categories of R-loops identified as specific to the RIS condition. In Fig. 8N, we show a class of R-loops that localize to genomic regions where RNase H1 fails to recognize and process these structures. These R-loops are formed in the presence of a G4 motif (“G-loop-like” structure) and coincide with regions of RPA32 hyperphosphorylation in RIS cells. This category accounts for approximately 5% of RIS-specific R-loops (Fig. 8N, Supplementary Fig. S8E).

In Fig. 8O, we present a second class of R-loops, also associated with G4 structures, that occur in genomic regions where RNase H1 is capable of binding but likely exhibits reduced ribonucleolytic activity. These regions are similarly marked by RPA32 hyperphosphorylation in RIS cells and represent approximately 43% of RIS-specific R-loops.

For comparison, Supplementary Fig. S8F shows a genomic region characterized by RPA32 hypo-phosphorylation in both H2B + RAS and RPA70 + RAS cells, where RNase H1 binding is comparable between the two conditions.

Overall, the genomic analysis reveals that R-loop accumulation in pre-RIS cells preferentially occurs in the following contexts:

- genomic regions with defective RNase H1 binding and impaired RPA binding (8.3% frequency);
- genomic regions with defective RNase H1 binding and RPA hyperphosphorylation (5.2% frequency);
- genomic regions with RNase H1 binding, RPA hyperphosphorylation, and likely impaired ribonuclease activity (43.1% frequency).

In summary, this analysis supports a model in which, in cells in the pre-RIS state, DNA:RNA hybrids characterized by RPA32 hyperphosphorylation are poor substrates for RNase H1. Impaired processing of these R-loops correlates with persistence of γH2AX signal and, in part, with altered replication fork dynamics.

## Discussion

OIS cells are known to accumulate persistent DNA damage, undergo extensive epigenetic remodeling [15], and were found in pre-malignant lesions and in chemotherapy-treated tumors [2–4]. Although the mechanisms that allow cells to bypass senescence and contribute to malignancy or chemoresistance remain incompletely understood, it is clear that both cell cycle regulators and factors involved in genomic and epigenetic stability play critical roles [6].

In our study, we observed increased levels of R-loops and G-loop-like structures in fibroblasts induced to express HRAS and prone to enter RIS compared with both models of RIS-bypass or normal proliferation. R-loops are known to accumulate near DSBs and serve as recruitment platforms for repair proteins, helicases, and nucleases [36, 39, 45, 49, 95]. However, unresolved R-loops can lead to genomic instability, particularly in highly transcribed regions [29, 96]. This functional duality reflects the heterogeneity in R-loop types [44], their formation and resolution kinetics [58, 62, 95] and the repair pathways involved [37, 39, 45, 97]. R-loops are essential for homologous recombination [98], but their processing, particularly during strand invasion [41], requires BRCA2-mediated resolution [38, 39]. Additionally, m5C modification within R-loops promotes HDR, while demethylation shifts repair toward PARP-1-dependent Alt-NHEJ [98].

We found that DNA:RNA hybrids accumulate aberrantly in RAS expressing cells at γH2AX-marked loci with impaired BRCA1 recruitment. This is partly due to defective RNase H1 recognition, resulting from compromised instruction by the RPA complex. RPA, a key regulator of ssDNA metabolism [55, 99], facilitates RNase H1-mediated R-loop processing in line with previous findings [48, 49, 100]. We identified the RPA70 groove as a docking site for RNase H1’s hybrid-binding domain, consistent with known interaction models [54, 55]. RPA supports homologous recombination by coating the 3′ ssDNA generated after DSB resection and enabling the handoff to RAD51—via BRCA1/BRCA2/PALB1 or, alternatively, RAD52—to initiate homology search and repair [84]. Our data raise the possibility that RPA–mediated potentiation of RNase H1 ribonuclease activity may also influence the efficiency of homologous recombination repair. Clarifying how this regulatory axis interfaces with HR–associated pathways will be an important focus of future studies. Importantly, in cells undergoing RIS this interaction is weakened due to DDR-induced hyperphosphorylation of RPA32, which alters RPA complex conformation and displaces RNase H1, similar to what has been observed with other RPA partners [88, 101]. A phosphomimic RPA32 mutant confirmed reduced RNase H1 activity and impaired R-loop resolution. The phosphorylation dependent control of RPA:RNase H1 activities represents a critical point, as the phosphorylation of RPA32 is tightly regulated [84]. During the G1/S transition, RPA32 is phosphorylated at S23 and S29 by CDK1 and CDK2 and subsequently dephosphorylated after mitosis [84]. In response to DNA damage, RPA32 becomes sequentially phosphorylated, with initial priming events at S29 and S33 mediated by ATR, followed by phosphorylation at T21 and S4/S8 by DNA-PK and ATM in the hyperphosphorylated form of RPA [84]. This hierarchical phosphorylation pattern indicates that damage-induced RPA32 hyperphosphorylation occurs downstream of earlier cell cycle- and ATR-dependent priming events [84, 102]. Phosphorylation of distinct RPA32 residues may differentially regulate homologous recombination in a context-dependent manner. In particular, while RPA phosphorylation at stalled replication forks has been associated with relief of replication stress [103], DNA-PK–dependent RPA32 phosphorylation has been reported to limit homologous recombination [104]. Our work supports a model in which HRAS–driven hyperphosphorylation of RPA, by dampening the ribonuclease activity of RNase H1 and promoting R–loop accumulation, contributes to a surveillance mechanism that halts cell-cycle progression and restrains neoplastic transformation.

Our findings are consistent with recent work from Knipscheer’s group [45], which identifies G–loops—DNA:RNA hybrids co–occurring with G4 structures—as intermediates in G4 resolution. The impaired processing of G–loops observed in RAS-expressing cells may therefore reflect defective G4 unwinding, potentially linked to altered RPA function [105]. At the same time, our evidence that RPA facilitates RNase H1 recognition and processing of G–loops does not exclude the contribution of additional pathways. Notably, Janscak’s group recently demonstrated that the helicase FANCJ can resolve G4 structures within G–loops [106], providing an alternative mechanism that, based on our data, may subsequently promote RNase H1–mediated removal of the associated R–loops. How the RPA complex integrates with, or coordinates alongside, helicase–driven G4 processing remains an open question for future investigation.

Finally, our model does not exclude alternative mechanisms of R-loop accumulation, such as disrupted RNA editing. Altered m6A or adenosine-to-inosine deamination has been observed in senescence and aging models [107]. m6A deposition at DSBs promotes RNase H1-mediated resolution [108], while ADAR2-mediated editing facilitates helicase-driven or RNase H2-mediated degradation [109]. These pathways may contribute to R-loop accumulation in OIS and warrant further investigation. Additionally, while our study emphasizes RPA’s role in regulating RNase H1, we acknowledge its broader functions in genome maintenance, including ssDNA protection, DNA repair, and DNA replication [84]. How these diverse activities of RPA are coordinated and integrated to regulate fundamental processes such as replication fork restart [110] remains to be clarified and will require further investigation in the future.

## Supplementary Material

gkag331_Supplemental_Files

## Data Availability

**Lead contact:** Further information and requests for resources and reagents should be directed to and will be fulfilled by the lead contact, E.D.G. (eros.digiorgio@uniud.it).

## Materials availability

All unique reagents generated as part of this study are available upon request from the lead contact with a completed Materials Transfer Agreement.

## Data and code availability

BRCA1 and RNAP2 Ser5 ChIP-seq from BJ-RAS, BJ + RAS and BJ + RAS/HDAC4 cells have been deposited at Gene Expression Omnibus under accession GEO: GSE287959.

γH2AX ChIP-seq from BJ-RAS, BJ + RAS and BJ + RAS/HDAC4 cells have been deposited at Gene Expression Omnibus under accession GEO: GSE216677.

DRIP-seq and G4 ChIP-seq from BJ-RAS, BJ + RAS and BJ + RAS/HDAC4 cells and ChIP-seq from BJ H2B + RAS and RPA70 + RAS cells have been deposited at European Nucleotide Archive under accession ENA: PRJEB101193.

Relevant bioinformatics analysis has been uploaded to the Galaxy platform and made available for consultation at the following link: https://usegalaxy.org.au/u/eros87/h/rip111224

Proteomics data have been deposited to the ProteomeXchange Consortium via the PRIDE partner repository (identifiers: PXD060652 and 10.6019/PXD060652).

Raw western blot and gel imaging data have been deposited at Mendeley data: 10.17632/t2858jvpwk.1

**•** This paper does not report original code.

**•** Any additional information required to reanalyze the data reported in this paper is available from the lead contact upon request.
