# Supplementary material for: RPA hyperphosphorylation hinders the resolution of R-loops and G-quadruplex-associated R-loops during RAS-driven senescence
Source: Nucleic Acids Res. 2026 Apr 13;54(7):gkag331. doi: 10.1093/nar/gkag331 (PMC13076219; doi:10.1093/nar/gkag331)
Supplement: gkag331_Supplemental_Files [file gkag331_supplemental_files.zip › Supplemental_documents_Cortolezzis_NAR2026_cleaned.docx]

**Supplementary documents**

**RPA Hyperphosphorylation Hinders the Resolution of R-Loops and G-Quadruplex-Associated R-Loops During RAS-Driven Senescence**

**Ylenia Cortolezzis^1^, Vanessa Tolotto^1^, Luca Triboli^1^, Raffaella Picco^1^, Miguel A. Soler^2^, Sara Fortuna^2^, Giacomo Bettin^3^, Francesca D’Este^1^, Enrico Carlassara^1^, Gabriele Magris^4^, Kyle M. Miller^5^, Alessandro Angelini^3^, Luigi E. Xodo^1*^, Eros Di Giorgio^1*^**

*Running title: Impaired RNase H1 loading by RPA leads to slower R-loop processing in senescence*

^1^ Laboratory of Biochemistry, Department of Medicine, University of Udine, Piazzale M. Kolbe 4, 33100 Udine, Italy

^2^ Dipartimento di Scienze Matematiche, Informatiche e Fisiche, University of Udine, Via delle Scienze 206, 33100 Udine, Italy

^3^ Department of Molecular Sciences and Nanosystems, Ca' Foscari University of Venice, Via Torino 155, 30172 Mestre, Italy.

^4^ Department of Agricultural, Food, Environmental and Animal Sciences, University of Udine, 33100 Udine, Italy

^5^ Department of Radiation Oncology, Emory University School of Medicine, Winship Cancer Institute, GA  30307 Atlanta, USA.

Correspondence: [eros.digiorgio@uniud.it](mailto:eros.digiorgio@uniud.it), [luigi.xodo@uniud.it](mailto:luigi.xodo@uniud.it)

Lead contact: [eros.digiorgio@uniud.it](mailto:eros.digiorgio@uniud.it)

Keywords: R-loops; RPA1; RPA2; RPA3; RNASE H1; oncogene-induced senescence; G-loops; DRIP-seq


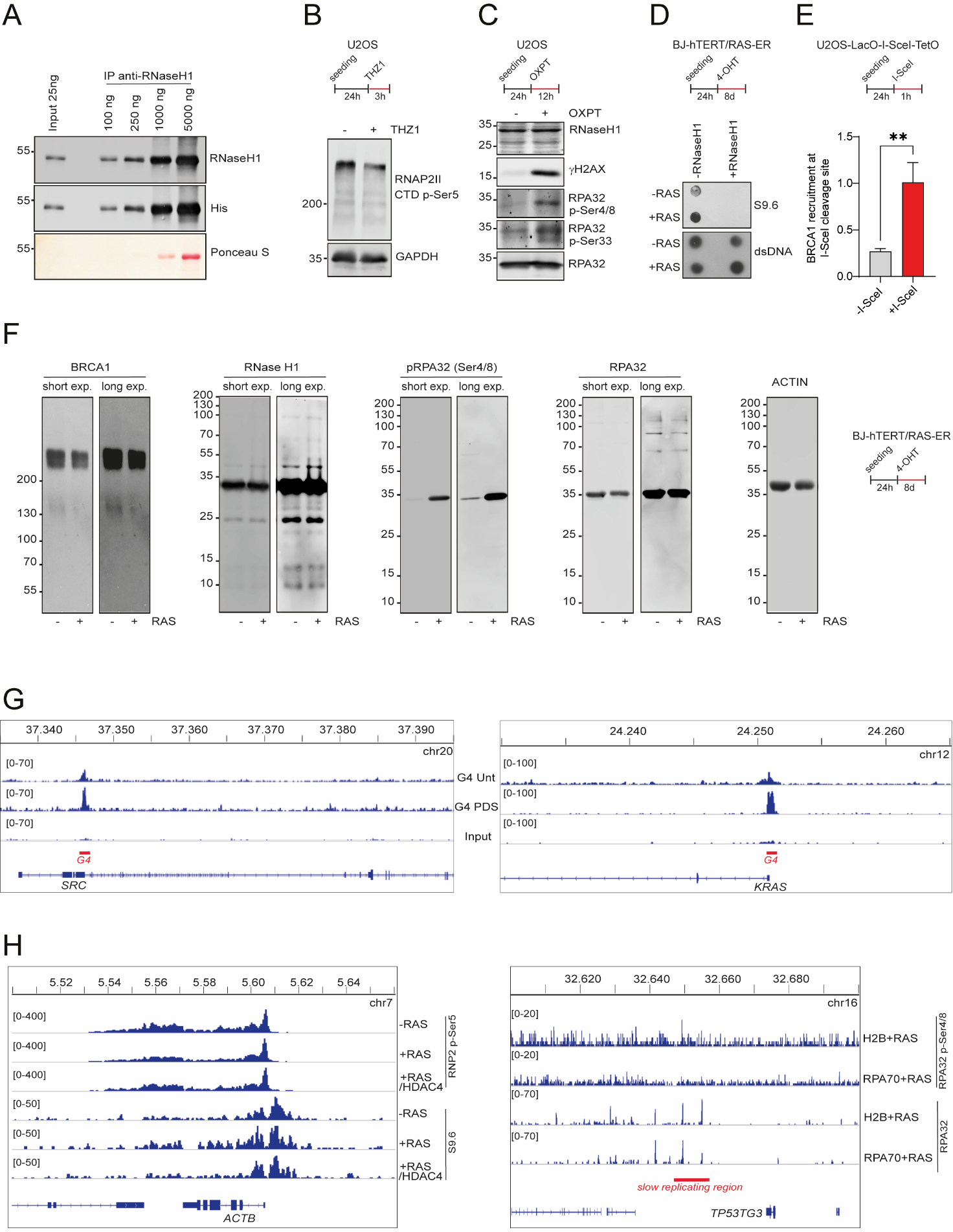


**Figure S1.** **Validation of antibodies used in ChIP-seq and DRIP-seq.** **A.**  Immunoblot analysis of recombinant His-tagged RNase H1 following immunoprecipitation with anti-RNase H1 antibody using increasing amounts of input protein, as indicated. Ponceau S staining is shown as loading control. Anti-His was used to confirm RNase H1 identity.

**B.** Immunoblot analysis of RNAPII CTD Ser5 phosphorylation (p-Ser5) in U2OS cells treated or not with 100 nM CDK7 inhibitor THZ1, as indicated. GAPDH was used as a loading control. **C.** Immunoblot analysis of RNase H1, γH2AX, phosphorylated RPA32 (p-Ser4/8 and p-Ser33), and total RPA32 in U2OS cells treated or not with 10 µM oxaliplatin (OXPT), as indicated. **D.** Dot blot analysis using S9.6 antibody to detect R-loops and anti-dsDNA antibody as loading control in BJ-hTERT/RAS-ER cells treated or not with 4-OHT, as indicated. Where indicated, samples where treated with 10 U RNase H1 to degrade R-loops. **E.** Quantification of BRCA1 recruitment at an I-SceI–induced double-strand break in U2OS-LacO-I-SceI-TetO cells treated or not with I-SceI, as indicated. Data are expressed as mean ± SD. p < 0.01. **F.** Immunoblot analysis using the indicated antibodies employed for ChIP-seq was performed on lysates collected from BJ-hTERT/RAS-ER cells expressing or not expressing RAS for 8 days, as indicated. The entire membrane is shown to highlight the presence of any potential non-specific bands. For BRCA1, proteins were resolved on a 6% acrylamide/bis-acrylamide gel, whereas all other proteins were separated on a 13% acrylamide/bis-acrylamide gel. For each antibody, both a short and a long exposure (to saturation) are shown to assess potential non-specific signals. Actin is provided as a loading control and was detected after stripping the membrane previously used for RPA32 detection. **G.** Genome browser tracks showing G4 enrichment at the *SRC* (left) and *KRAS* (right) loci detected using BG4-specific pull-down in PANC-1 cells under untreated (Unt) or pyridostatin-treated (PDS, 1 µM) conditions. Input tracks are shown as controls. Well-defined G4s, characterized from a molecular and biophysical standpoint, are indicated. **H.** Genome browser tracks showing: (left) RNAPII p-Ser5 occupancy and R-loop levels (S9.6) at the *ACTB* locus in BJ-hTERT expressing or not HRAS (RAS) and HDAC4, as indicated. A well-defined R-loop is evident at the *ACTB* TSS; (right) RPA32 p-Ser4/8 and total RPA32 occupancy at the *TP53TG3* locus in H2B+RAS or RPA70+RAS cells. The slow-replicating region is indicated (GSE299123). Co-localization of pRPA32 and RPA32 is evident at the identified slow-replicating region exclusively in H2B+RAS cells, but not in RPA70+RAS cells.


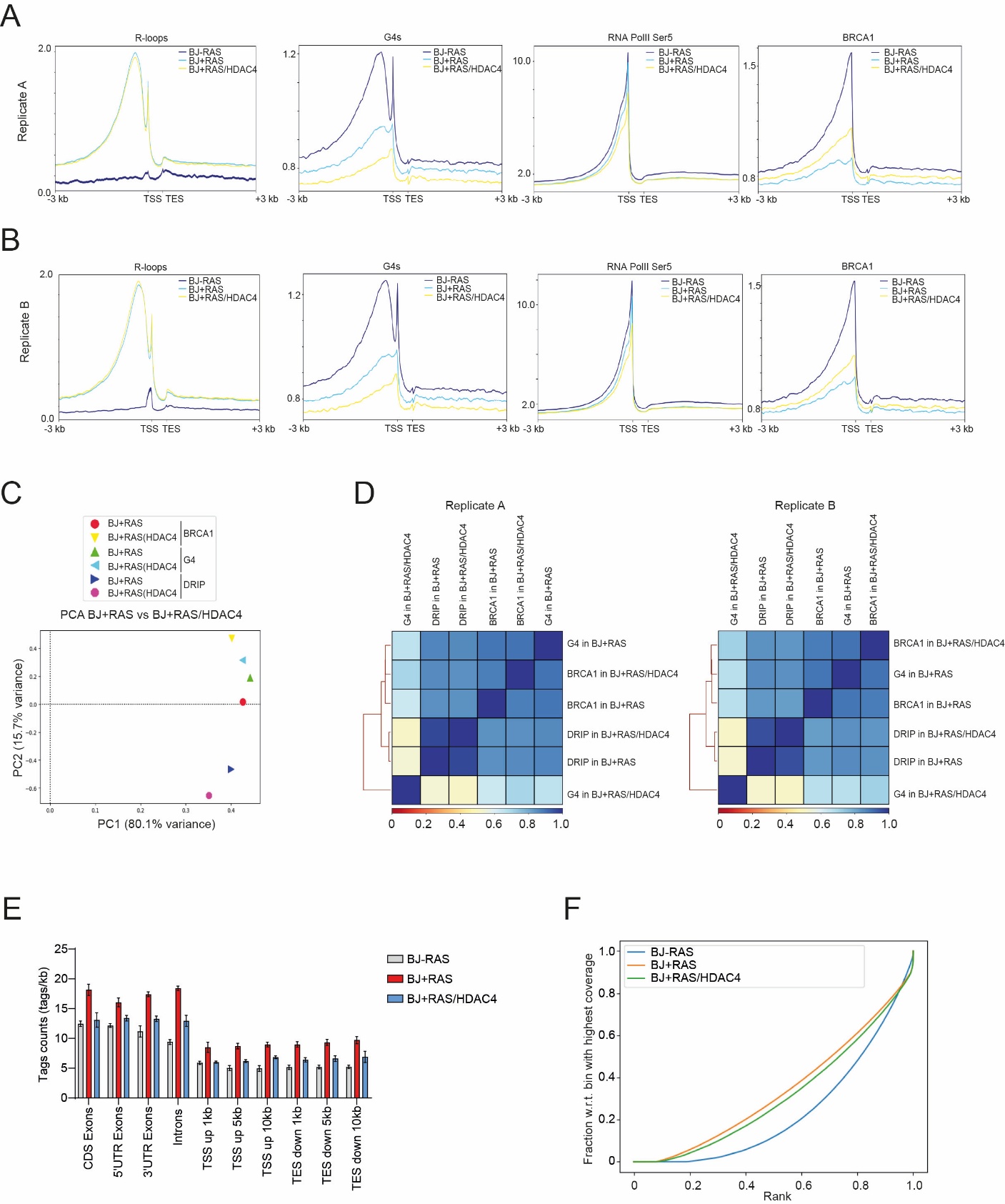


**Figure S2. Characterization of ChIP-seq and DRIP-seq.** **A, B.** Metaplots of R-loops, G4, RNA PolII Ser5 and BRCA1 in respect to TSS and TES. GRCh38 genome assembly was used. **C.** PCA plot of the indicated samples in the indicated cellular conditions. Average coverages were computed over 10 kb bins, from bigWig files using multiBigwigSummary. **D.** Spearman correlation plot generated using the bigWig files corresponding to each sample, as indicated in the legend. Average coverages were computed over 10 kb bins, from bigWig files using multiBigwigSummary. This was then used with plotCorrelation to make a heatmap of Spearman correlation coefficients. Hierarchical clustering was performed using Euclidean distance to group samples based on similarity in their coverage profiles. **E.** Histogram showing the distribution of γH2AX+ read counts across the identified regions (TSS, TES, promoters, exons, introns) in the three cellular conditions compared at day 8 of HRAS expression. **F.** Fingerprint analysis of γH2AX ChIP-seq data. Cumulative read distribution plots show a more uniform genome-wide distribution of γH2AX signal in RAS-expressing cells compared with normally proliferating BJ cells, consistent with broad γH2AX accumulation in RIS. HDAC4-expressing cells display an intermediate profile, indicating partial restoration toward a more focal γH2AX distribution.


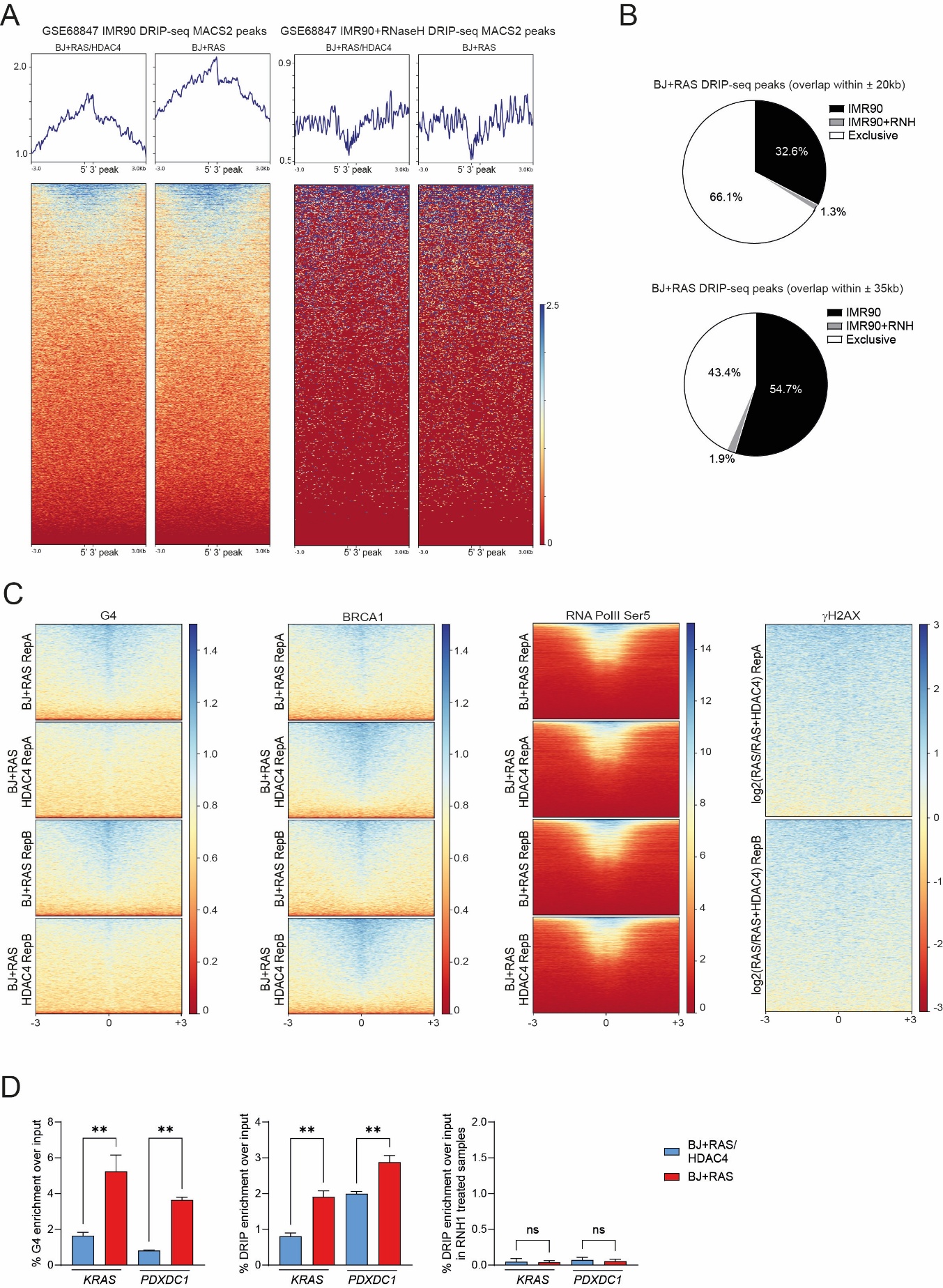


**Figure S3. Comparative analysis of R-loops and G-quadruplexes. A.** Heatmap and profile of DRIP-seq signal in BJ+RAS and BJ+RAS/HDAC4 cells within 6 kb around the DRIP-seq peaks found enriched in IMR90 (Etoposide-treated) and RNase H-treated IMR90, as indicated. IMR90 data were retrieved from GEO: GSE68847. **B.** Venn diagrams showing the percentage of peaks found to be exclusive to the BJ+RAS condition that overlap with peaks identified in the reference study using IMR90 cells treated with either Etoposide or RNase H, across two defined genomic intervals. The overlap is substantial with the former, but markedly weaker with the RNase H treatment, as expected. **C.** Heatmaps of G4, BRCA1, RNA Pol II Ser5, and γH2AX signals within a 6 kb window centered on R-loop peaks exclusive to the BJ+RAS condition. **D.** qPCR validation of G4-seq and DRIP-seq of the indicated samples and genomic loci. Mean ± SD; n = 3. *P< 0.05, **P< 0.01. Dunn's Multiple Comparison Test was applied to indicated comparisons.


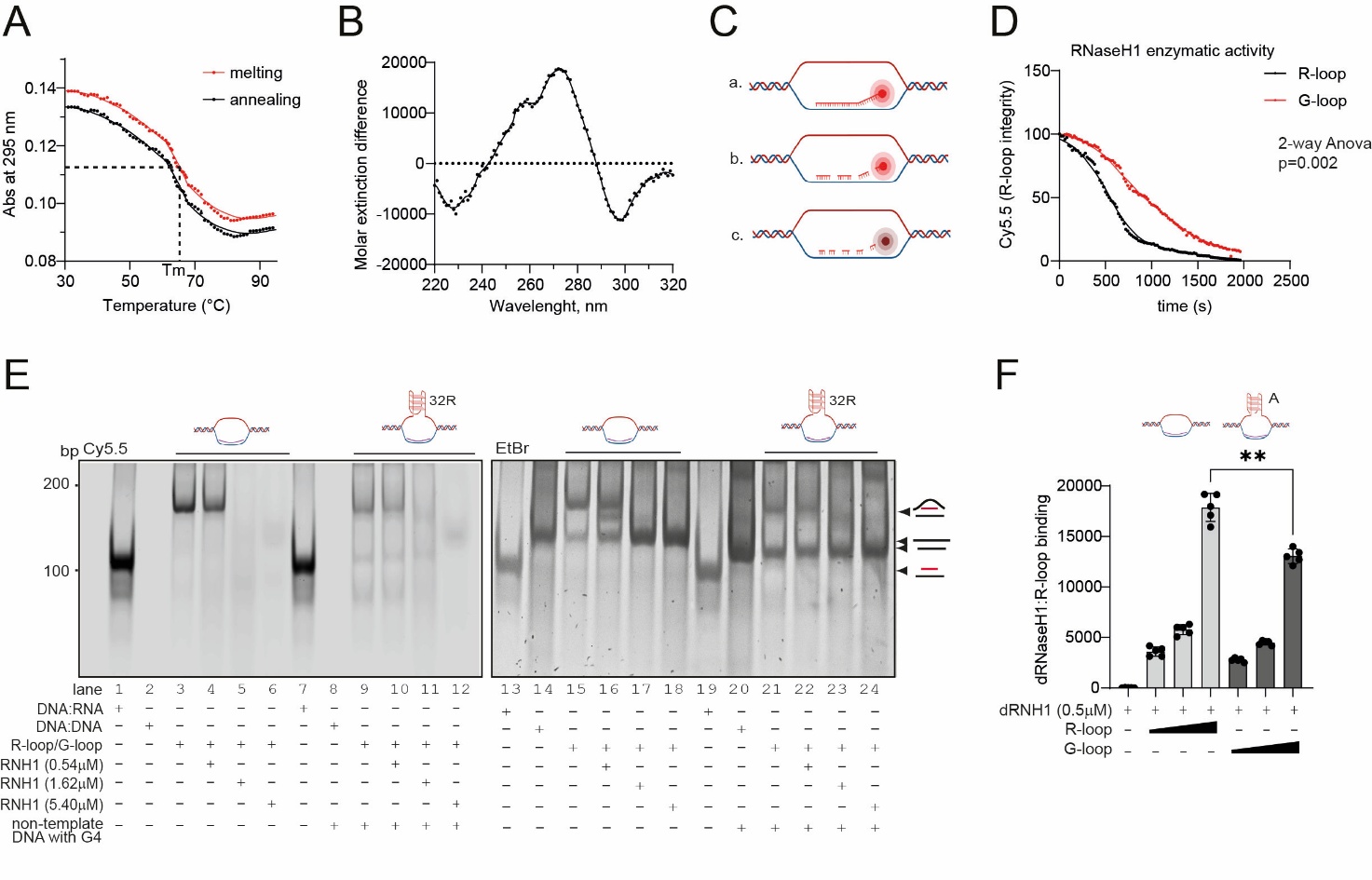


**Figure S4. Molecular characterization of R-loops and G-loops. A.** Melting temperature (Tm) curve measured by absorbance at 295 nm for a DNA oligonucleotide containing motif A at a concentration of 1 μM, recorded during both the heating (melting) and cooling (annealing) phases. **B.** Thermal difference spectrum resulting from the subtraction of UV absorbance spectra (expressed as molar extinction, M−1 cm−1) at 25°C from the spectrum at 95°C. **C.** Schematic of the RNase H1 ribonucleolytic kinetics assay. a. At time zero, the Cy5.5 fluorescence of a synthetic DNA:RNA hybrid is quantified, in which the RNA strand is conjugated to a Cy5.5 fluorophore via a 5′ phosphodiester bond. The addition of RNase H1 initiates the kinetic reaction. b. Endoribonucleolytic cleavage leads to the generation of RNA fragments, as illustrated in the schematic and visible in the electrophoretic profiles shown in Fig. 3E, 4I, 4J, and 6D. c. Loss of fluorescence at the 5′ end of the RNA may result either from exonucleolytic activity that cleaves the phosphodiester bond linking the fluorophore, or from fluorescence quenching in endoribonucleolytic fragments generated. As a control, no significant fluorescence perturbation was observed by using deadRNase H1. **D.** Sigmoidal kinetics of ribonucleolytic activity on two equimolar substrates, highlighting a lower Km and higher kcat for the synthetic R-loop compared to the G-loop. **E.** The in vitro assay was performed by incubating Cy5.5-labelled R-loops, G-loops containing the G4 motif KRAS 32R or DNA:RNA duplex (Cy5.5 labelling at 5’ of RNA) with increasing amounts of RNase H1. Native gel electrophoresis was performed to separate the various species of nucleic acids, which were schematized on the side. The fluorescence of the Cy5.5 labelled RNAs was acquired with the fluorescence reader and then the gel was stained with EtBr to detect the DNA duplexes with the transilluminator. G4 in displaced DNA strand was present in lanes 8-12 and 20-24. **F.** Increasing amounts (150 pmol, 300 pmol, 600 pmol) of synthetic R-loop or G-loop (Cy5.5 labelled at 5’ of RNA) were incubated for 3h with a fixed amount of His-deadRNase H1 (dRNase H1) immobilized on magnetic streptavidin beads in RNase H binding buffer (5 mM Hepes, 10 mM NaCl, 30 mM KCl). After washing, the amount of DNA:RNA hybrids recovered—and thus bound by dRNase H1—was estimated by measuring Cy5.5 fluorescence.


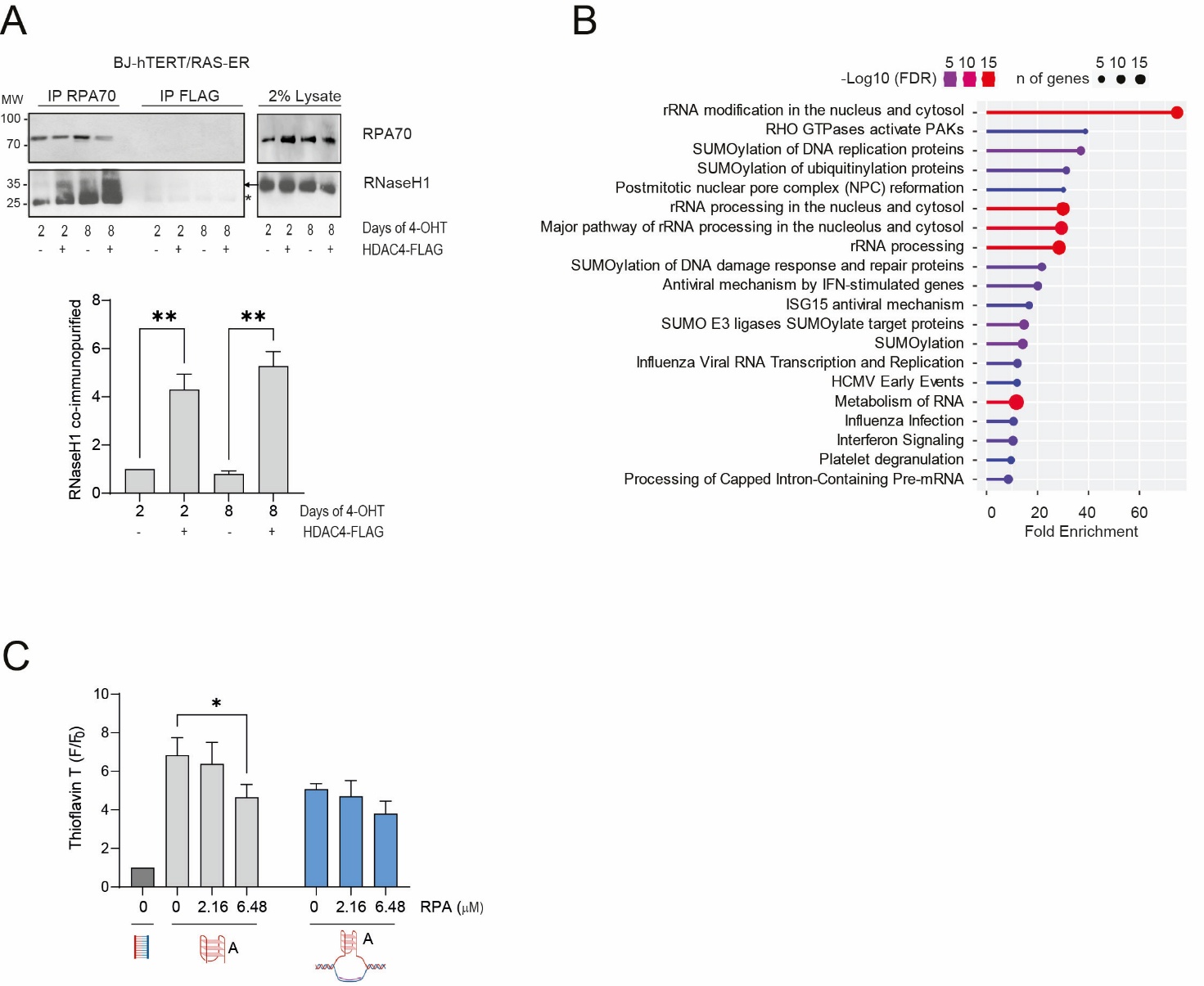


**Figure S5. Modulation of RPA–RNase H1 activity in different cellular states. A.** Co-IP experiment in BJ-hTERT/RAS-ER cells expressing or not HDAC4 as indicated, treated or not with 4-OHT to induce HRAS expression as indicated. Native lysates were immunoprecipitated with 1 μg anti-RPA70 antibody or anti-Cherry antibody as a control. The asterisk indicates the light chain of the antibody used in the IP, which appears more intense than the IgG control—likely due to different isotypes (IgG2a for RPA70, IgG1 for FLAG). The arrow marks the specific RNase H1 band. Histogram reporting quantification is provided. Mean ± SD. **p< 0.01. Pairwise t-test was applied to indicated comparisons. **B.** Functional categories associated with the 48 hits (RPA1 interactors, Supplementary Table S6) found associated in proliferating BJ-hTERT cells but not in those expressing RAS. Functional enrichment was performed using g:Profiler (RRID: SCR_006809). Pathways are ranked by fold enrichment; dot color represents –log10(FDR), and dot size indicates the number of genes associated with each pathway. **C.** Increase in Thioflavin T fluorescence following labeling of equimolar amounts of DNA G4 or G-loop structures (both containing G4 motif A) in the presence of increasing concentrations of the RPA complex. A DNA duplex not containing G4 is used as a control. Only partial unwinding of the G4 structure was observed. Mean ± SD. *p< 0.05.


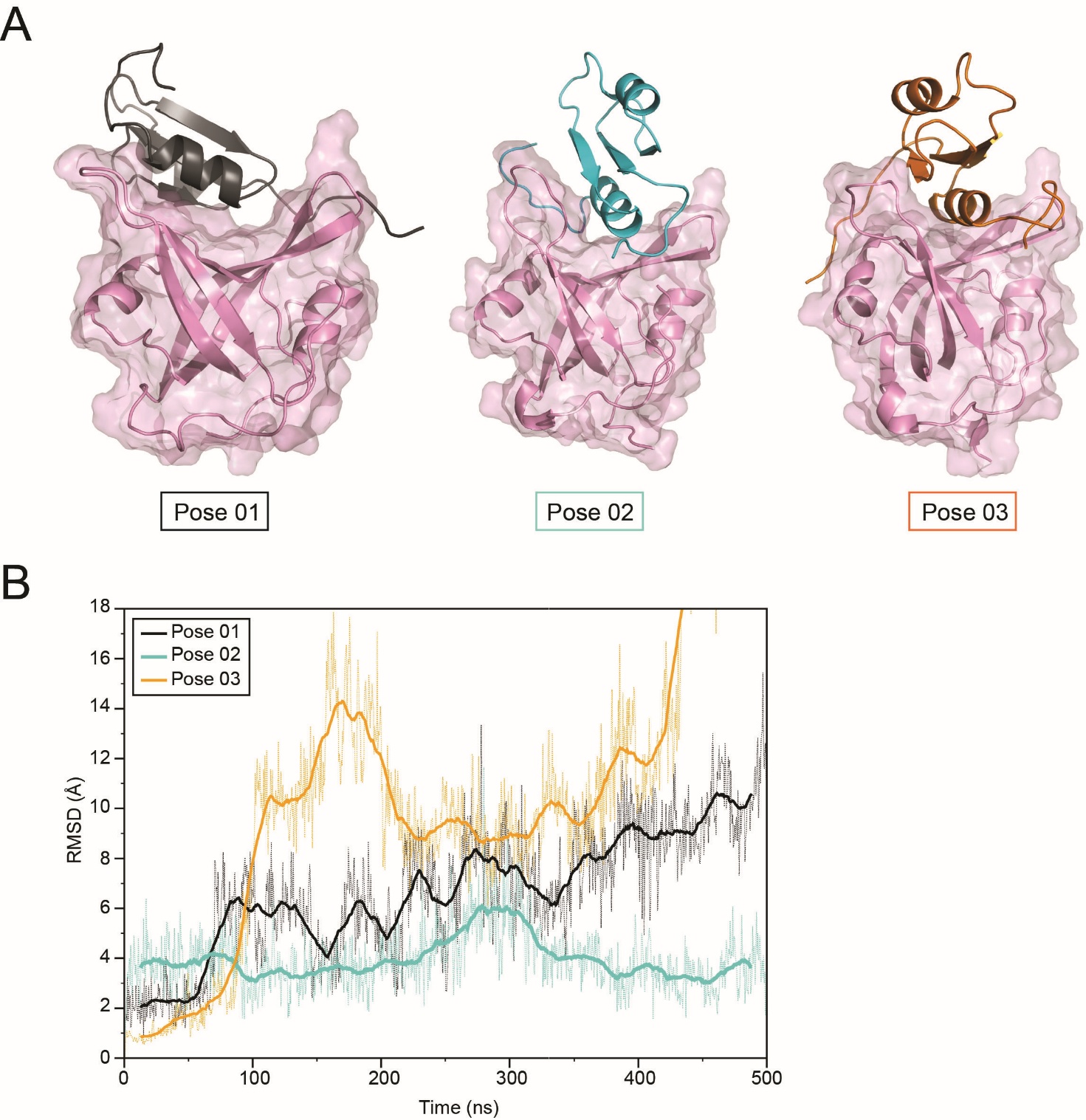


**Figure S6. Docking and molecular dynamics simulations of the RPA–RNase H1 complex. A.** Graphical representation of the three main binding modes found during the docking exploration. The displayed configurations were obtained after the MD simulation protocol for exploring the most probable binding conformation for the disordered RNase H1 regions. **B.** Root mean square deviation (RMSD) analysis of the backbone of the RNase H1 hybrid-binding domain relative to the docked initial complex along the molecular dynamics (MD) trajectories. The backbone of the RPA70 OB domain was used for structural alignment. Increasing and unstable RMSD values indicate progressive deviation from the initial configuration and are therefore indicative of reduced stability of the binding conformation.


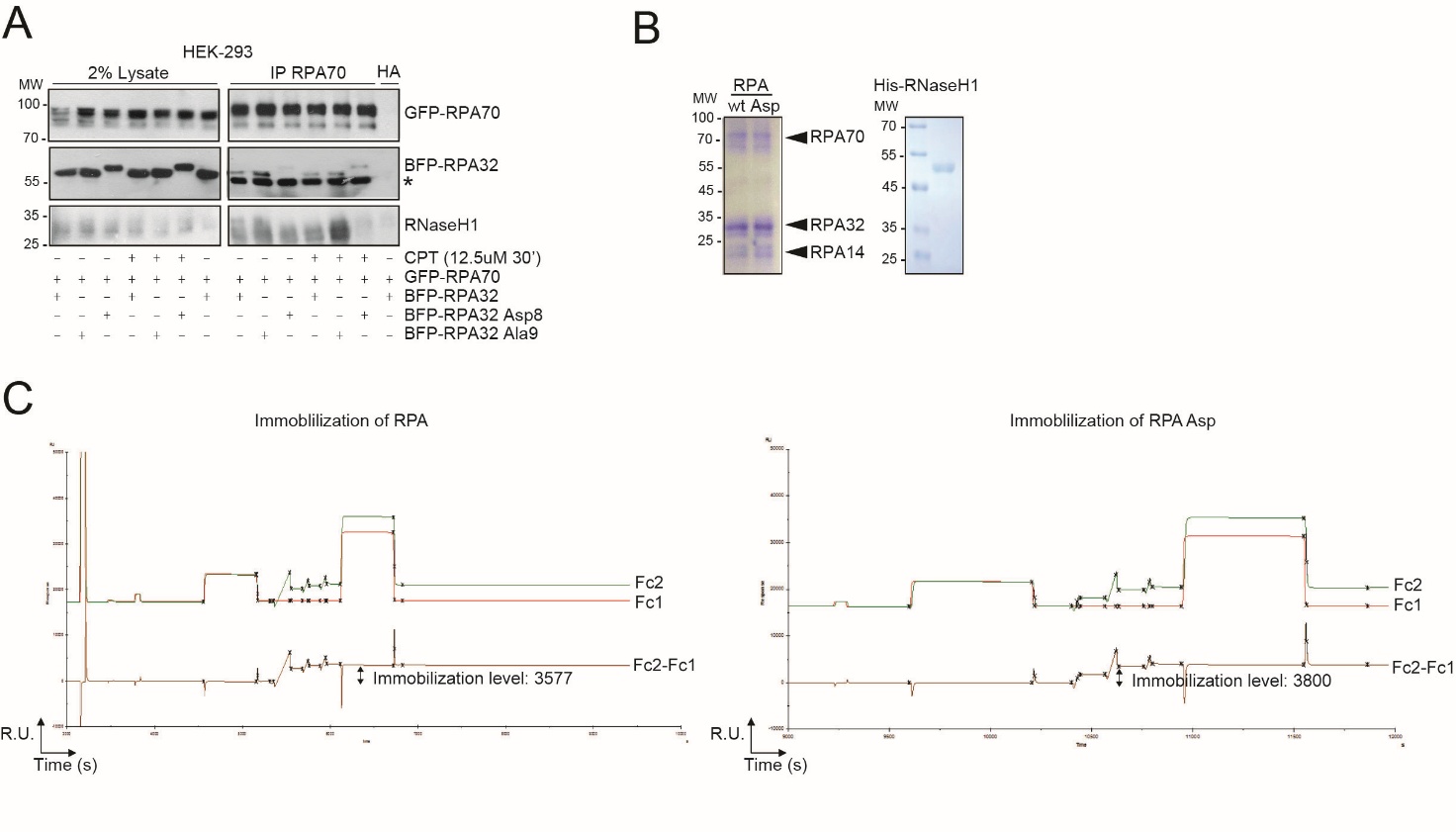


**Figure S7. RPA purification and surface plasmon resonance (SPR) experimental set-up. A.** HEK-293 cells were co-transfected with plasmids expressing RNase H1-FLAG, BFP-RPA32 (wild type, phosphodead (Ala9) or phosphomimetic (Asp8)) and GFP-RPA70, as indicated. Cells were harvested after 30’ of treatment with CPT, as indicated. Immunoprecipitation was performed by using 1 μg of anti-RPA70 antibody. 1/50 total lysate was included as input. The asterisk indicates a non-specific signal detected with the anti-RPA32 antibody. **B.** Blue Coomassie staining of the indicated purified recombinant proteins and separated by SDS-PAGE. **C.** The SPR sensorgrams represent the process of immobilization of RPA and RPA Asp. Equivalent immobilization efficiency was achieved in the two cases.


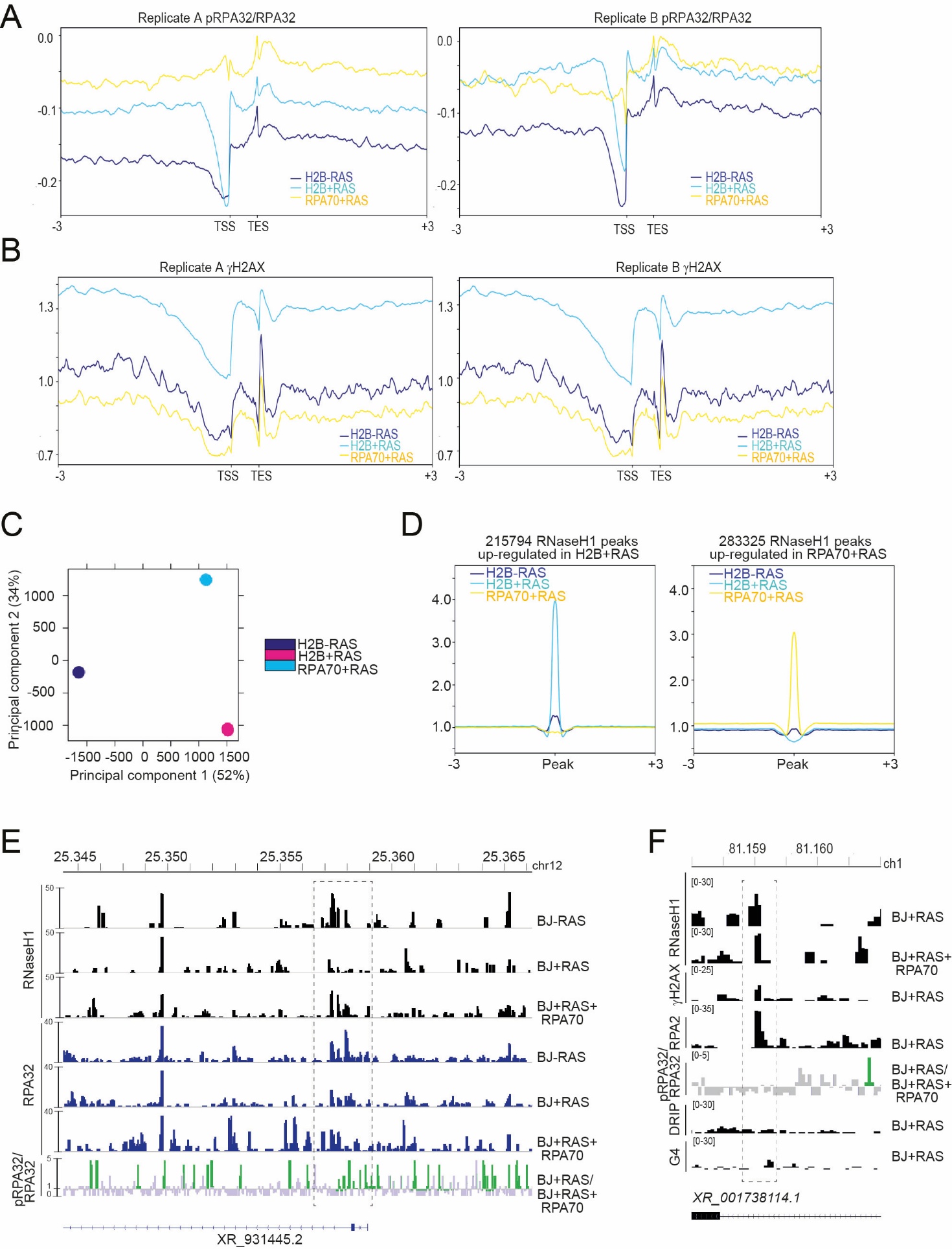


**Figure S8. Integrative genome-wide analysis of ChIP-seq and DRIP-seq data. A, B.** Metaplot of pRPA32/RPA32 (expressed as ratio) and γH2AX reads in the indicated samples within 3 kb from TSS and TES. **C.** PCA plot illustrates the diversity among the three cellular conditions compared, based on RNase H1 signal profiles. **D.** Metaplots of RNase H1 ChIP-seq levels in the indicated cells, within a genomic interval of 6 kb around the center of RNase H1 peaks enriched in the H2B+RAS or RPA70+RAS conditions. **E.** Genomic track of RNase H1, RPA32 and pRPA32/RPA32 signals around a class of R-loops that localize to genomic regions where RNase H1 fails to recognize, bind, and process these structures in pre-RIS condition. This genomic region (highlighted) coincides with RPA32 hyperphosphorylation in RIS cells. **F.** Track of a genomic region characterized by RPA32 hypo-phosphorylation in both H2B+RAS and RPA70+RAS cells, where RNase H1 binding is comparable between the two conditions and R-loops were not detected in pre-RIS cells.

**Table S1.** DNA and RNA oligos used in this study.

**Table S2.** Antibodies used in this study.

**Table S3.** Gene associated to R-loops specific for pre-RIS condition (association rules: Proximal -5kb, +1kb; Distal: 1kb).

**Table S4.** Genomic coordinate of R-loops characterizing BJ+RAS cells (hg38).

**Table S5.** Table of the167 interactors of RPA70 identified in BJ-hTERT proliferating cells by MS.

**Table S6.** Table of 48 out of the 167 interactors reported in Table S5 that were found to be complexed with RPA70 in BJ-hTERT proliferating cells but not, or significantly less, in BJ-hTERT/*HRAS^G12V^* undergoing OIS (log2(fold-change)>0.5, p<0.1)).
